# Carbides and Nitrides of Zirconium and Hafnium

**DOI:** 10.3390/ma12172728

**Published:** 2019-08-26

**Authors:** Sergey V. Ushakov, Alexandra Navrotsky, Qi-Jun Hong, Axel van de Walle

**Affiliations:** 1 Peter A. Rock Thermochemistry Laboratory and NEAT ORU, University of California at Davis, Davis, CA 95616, USA; 2 School of Engineering, Brown University, Providence, RI 02912, USA

**Keywords:** zirconium carbide, hafnium carbide, zirconium nitride, hafnium nitride

## Abstract

Among transition metal carbides and nitrides, zirconium, and hafnium compounds are the most stable and have the highest melting temperatures. Here we review published data on phases and phase equilibria in Hf-Zr-C-N-O system, from experiment and ab initio computations with focus on rocksalt Zr and Hf carbides and nitrides, their solid solutions and oxygen solubility limits. The systematic experimental studies on phase equilibria and thermodynamics were performed mainly 40–60 years ago, mostly for binary systems of Zr and Hf with C and N. Since then, synthesis of several oxynitrides was reported in the fluorite-derivative type of structures, of orthorhombic and cubic higher nitrides Zr_3_N_4_ and Hf_3_N_4_. An ever-increasing stream of data is provided by ab initio computations, and one of the testable predictions is that the rocksalt HfC_0.75_N_0.22_ phase would have the highest known melting temperature. Experimental data on melting temperatures of hafnium carbonitrides are absent, but minimum in heat capacity and maximum in hardness were reported for Hf(C,N) solid solutions. New methods, such as electrical pulse heating and laser melting, can fill the gaps in experimental data and validate ab initio predictions.

## 1. Introduction

**Figure 1:**
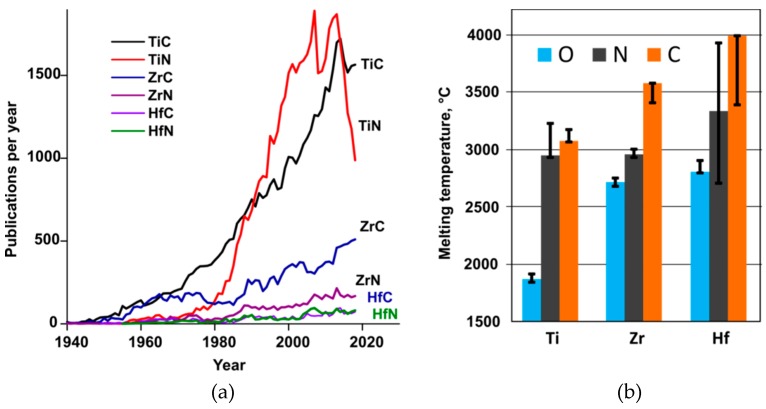
(**a**) Number of publications per year on carbides and nitrides of titanium group transitional metals (1940–2018 Chemical Abstract database). (**b**) The preferred values for melting temperatures of Ti, Zr, and Hf carbides and nitrides with NaCl-type structure. See Section 4 for references. The “error bars” indicate the range of previously reported values for melting temperatures from Berg et al. [7].

The carbides and nitrides of zirconium and hafnium provide a reservoir of very effective refractory materials, including some compounds that have been predicted to have the highest melting points [1]. Ti, Zr, and Hf belong to group IVb of transition metals. These so-called titanium group elements form the most stable nitrides and carbides known [2]. They share NaCl-type (rocksalt) structure, high thermal and electrical conductivity, strength, and hardness. Titanium carbide and carbonitrides have been used as cermets constituents since 1970; most of the produced tools for metal cutting are coated with multilayer TiN-TiCN-TiC coatings, applied through physical or chemical vapor deposition processes. These coatings reduce wear through increased hardness and lowering frictional welding at high operating speed [3]. For the last three decades, more than 1000 papers have been published every year on Ti carbides and nitrides (Figure 1). Although melting temperatures for carbides and nitrides of zirconium and hafnium are higher than those for Ti, they are studied less and their applications are currently limited. 

There are more publications on ZrC per year than for ZrN, HfC, and HfN combined. This is driven by the research on ZrC for nuclear-related applications such as barrier coatings for nuclear rocket reactors [4] and replacement of SiC in tri-isotropic (TRISO) fuel for high temperature nuclear reactors [5]. Nitrides of actinides are studied as advanced nuclear fuels compatible with liquid metal coolant, and ZrN is a primary matrix candidate for Pu based nuclear fuel [6]. 

Due to high neutron absorption of Hf, its carbides and nitrides did not receive as much attention as zirconium counterparts for nuclear-related applications; however, they have a high potential for advanced solar energy-related applications. Partially oxidized hafnium carbide coatings showed high spectral selectivity as absorber coating for concentrated solar power systems [8], and hafnium nitride as an absorber for hot carrier solar cells [9]. For polymer electrolyte membrane fuel cells, ZrN coating was shown to significantly increase corrosion resistance and hydrophobicity of stainless steel for replacement of highly brittle graphite-based bipolar plates [10]. 

Zirconium and hafnium nitrides are also attractive hard coatings for decorative and architectural glass applications [11]. They have golden-green and yellow-green colors, which can be fine-tuned by nitride stoichiometry and by alloying with Ti and Al nitrides and carbides [12]. Zirconium nitride coated tools and scalpel blades are commercially available and are considered to be superior for Al cutting applications and have high biocompatibility.

Recently, Zr and Hf oxynitrides made it into the playbook of the semiconductor industry since current leakage through thermally grown SiO_2_ thin films forced a search for a suitable replacement material with a higher dielectric constant. Amorphous Zr and Hf oxynitrides are researched as gate dielectrics in integrated circuits [13,14,15,16]. Hafnium oxynitride also forms on SiN-HfO_2_ interface, where HfO_2_ used as gate dielectric and SiN as a diffusion barrier on Si [17]. 

Finally, one should mention the application which funded most of the research on refractory carbides, nitrides, and borides at high temperature: it is their use as constituents of ultra high temperature ceramics, studied since the 1950s for thermal protection systems for atmospheric reentry and, more recently, for sharp leading edges of hypersonic missiles [18,19]. 

Carbides and nitrides of Zr and Hf were previously reviewed together with other transition metals in monographs by Goldschmidt [20] and Storms in 1967 [21], Toth in 1971 [22], Pierson in 1996 [23], and in a collection of papers edited by Freer in 1990 [24], Samsonov in 1995 [25], Oyama in 1996 [26], Gogotsi and Andrievski in 1999 [27]. Catalytic properties were reviewed by Levy in 1977 [28] and by Ham and Lee in 2009 [29]. Monograph by Turchanins 1991 [30] presented in details 20-year of original experimental work on the thermodynamics of refractory carbides and carbonitrides, some of which were previously published in English [31,32,33,34], but the monograph is only available in Russian. Samsonov, Upadhyaya, and Neshpor published in Russian a monograph on carbides in 1974 [35]. The updated and extended monograph was published by Upadhyaya 1996 [36] in English. This encyclopedic, 500 pages compendium also covers binary and ternary systems with boron and nitrogen and based on more than 2000 original research reports published before 1980. During the last 20 years, Lengauer et al. [37,38,39,40,41] provided comprehensive reviews and new experimental data on solid state properties of Ti group carbonitrides. Finally, the second volume of Shabalin’s (2019) [42] monograph on ultra-high temperature materials provides an exhaustive 350 pages reference on chemical and physical properties of hafnium and zirconium monocarbides. 

In this paper, we review thermodynamic data available from experiment and computations for the Zr-C-N and Hf-C-N systems. Our focus is on rocksalt (δ-phase) (oxy)carbonitrides: monocarbide and mononitrides of Zr and Hf, their solid solutions, and oxygen solubility limits. They are the only compounds in these systems that melt without decomposition and are traditionally considered most interesting for applications. We will briefly touch on non-metallic higher nitrides: Zr_3_N_4_ and Hf_3_N_4_ attracted a lot of attention due to the change from insulator to metallic conductivity upon nitrogen loss [43,44,45,46,47] and their hardness in Th_3_P_4_-type structure, stable at high pressure, but also synthesized as coatings at ambient pressure. 

First, we briefly overview synthesis methods, established binary phase diagrams, and reported and predicted phases. Then, we review the structure and thermochemistry of rocksalt (oxy)carbonitrides and list recommendations for future experimental and computational works.

## 2. Synthesis Methods

Zirconium and hafnium carbides and nitrides can be synthesized by direct reaction of metal or metal hydride powders with graphite or N_2_. The reactions are strongly exothermic and can be used in a self-propagated high temperature synthesis (SHS) process [48]. The reaction of oxides with graphite (carbothermal process) is widely used for industrial synthesis of bulk carbide powders. Nitrides can also be synthesized from oxides [49] by reaction with nitrogen or ammonia, usually in the presence of carbon (carbothermal nitridation) [26,50]. The traditional preparation routes were reviewed in monographs by Toth [22] and Storms [21]. The enthalpies and entropies for the selected reactions are presented in Table S1.

Recent research efforts in the synthesis of carbides and oxycarbides were aimed at the production of fine powders. For ZrC, this was achieved using polymer precursors or zirconia sol-sucrose mixture, or by decomposition of zirconium-based metal-organic frameworks (MOFs) [51]. ZrN powders with submicron size were also recently synthesized by reduction of ZrO_2_ with Al metal in N_2_ atmosphere [52] using CaCO_3_ addition to form HCl- soluble calcium aluminate and zirconate phases. This method was inspired by a similar route earlier employed for TiN [53], and many syntheses approaches established for TiN and TiC can be adapted for Zr and Hf carbides and nitrides.

The variety of chemical and physical vapor deposition (CVD and PVD, respectively) methods for refractory carbides was reviewed by Pierson (1996) [23], although more research was focused on titanium carbonitrides. Carbides, nitrides, and carbonitrides of Zr and Hf can be prepared by CVD at 900–1200 °C from metal chlorides (ZrCl_4_ or HfCl_4_) and nitrogen or ammonia as a source of nitrogen or hydrocarbons as a carbon source. These CVD reactions usually proceed in the presence of hydrogen. HfC deposition by CVD was demonstrated using methane (CH_4_), propane (C_3_H_8_), propene (C_3_H_6_), or toluene (C_7_H_8_) and methyl chloride (CH_3_Cl). Apart from TiCl_4_, hafnium, and zirconium, chlorides are solid at room temperature, but can be synthesized in situ by metal reaction with Cl or HCl at 500–600 °C. Carbide and nitride coatings can also be produced by PVD methods, e.g., reactive evaporation of metals in nitrogen, ammonia or hydrocarbon atmospheres, molecular beam evaporation, sputtering, and ion plating [54]. 

Zr and Hf carbides and nitrides form continuous solid solutions in the rocksalt structure and can contain substantial amounts of oxygen. This makes it possible to obtain different compositions and tune the properties by slight adjustments of synthesis conditions; however, it also makes it challenging to reproducibly synthesize pure compounds with well-defined stoichiometry. Carbides obtained by carbothermal reaction from oxides are known to retain some oxygen [55]. Nitrides from carbothermal nitridation will contain both oxygen and carbon impurities [50]. Synthesis from metal hydrides is often considered most appropriate for producing pure compounds; however, hydrogen can also be incorporated in rock-salt structure, e.g., formation of the solid solution ZrC_0.67_H_0.33_ has been reported [56]. The continuous solid solutions, coupled with nonstoichiometry and technical difficulties analyzing light elements, are the reasons for large scatter in structural data and properties attributed to nominally same compositions from different synthesis routes as discussed below. 

## 3. Phase Diagrams 

The binary systems of zirconium and hafnium with nitrogen and carbon were studied experimentally from circa 1000 °C to liquidus temperatures and most of the systematic studies of phase equilibria were performed before 1970. New experimental results on melting temperature determination and thermodynamic properties of single phases were published since then [61,62,63,64,65,66], and an entirely new stream of thermodynamic data has emerged from ab initio computations [1,67,68,69].

The state of the art calculation of phase diagrams (Calphad) method [70,71,72,73] allows for the calculation of phase equilibria in multicomponent systems above room temperature at atmospheric pressure, providing that Gibbs free energy functions for all phases from room temperature to the melting point and above can be derived from observed phase equilibria and thermodynamic data. In Calphad assessments, ab initio computational results are treated on the same footing as experimental data. 

**Figure 2:**
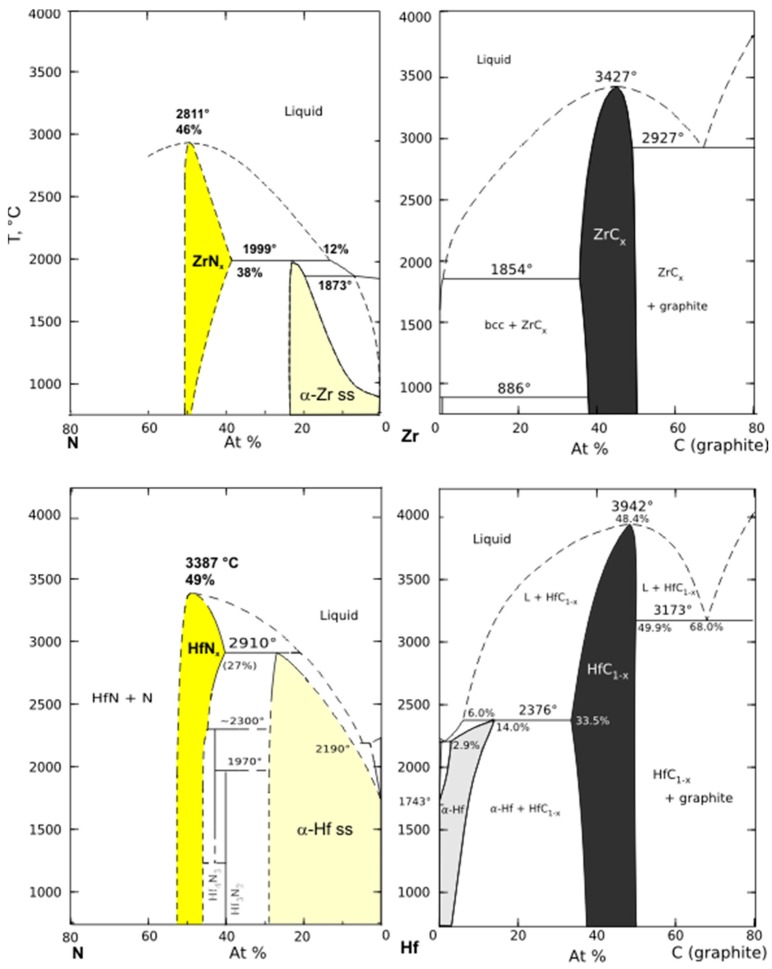
Binary phase diagrams for condensed system of Zr and Hf with graphite and nitrogen (at 1 atm pressure), redrawn after: (Zr-N)—Ma et al. (2004) [57]; (Zr-C)—Gullerment (1995) [58]; (Hf-N)—Okamoto (1990) [59]; (Hf-C)—Bittermann and Rogl (1997) [60].

The availability and accuracy of experimental data for metals [74,75] and a unified set of unary data [76] have enabled the great practical success of the Calphad approach for the engineering of metal alloys [77,78]. Calphad application to carbides, nitrides, and oxides is hampered by lack of data and complexity of structures. The latest binary phase diagrams for Hf and Zr systems with nitrogen and carbon are shown in Figure 2. Thermodynamic assessment using the Calphad method was performed on Zr-C, Hf-C, and Zr-N systems by Guillermet in 1995 [58], Bittermann and Rogl in 1997 [60] and Ma et al. in 2004 [57], respectively. No Calphad assessment of Hf-N system is available. 

### 3.1. Zr-C, Hf-C, and Zr-Hf-C 

Calphad assessment of Zr-C system by Guillemet [58] relied on phase equilibria data from Sara [79] and Rudy [80], selected values for enthalpies of formation and high temperature heat contents for ZrC_0.69_-ZrC_0.99_, and low-temperature heat capacity and entropy for ZrC_0.96_. Gullermet [58] found significant discrepancies between different reports on high temperature heat capacities of Zr carbides and did not use them in his optimization. Since then, a review on ZrC properties was published by Jackson and Lee [81], which provides a comprehensive compilation of thermodynamic data on ZrC system including heat capacities as a function of C/Zr ratio. Gullermet’s [58] assessment of the Zr-C system was used in a number of later Calphad assessments of ternary systems [55,82]. 

Calphad treatment of Hf-C system by Bittermann and Rogl [60] follows the version of the experimental phase diagram presented by Rudy (1969) [80]. Formation enthalpy and high temperature heat content for HfC_1−x_ was included in the optimization. The calculated phase diagram reproduces well most of Rudy’s [80] experimental points, with the exception of liquidus boundaries on Hf-HfC side. Bittermann and Rogl [60] attribute this difference to inherent uncertainties in the Pirani method [83] used by Rudy to obtain liquidus data. In a 2001 review of Hf-C system by Okamoto [84], Bittermann and Rogl’s [60] assessment is accepted as a better representation of the system than original work by Rudy [80]. 

Bitterman and Rogl [85] also performed a thermodynamic assessment of the Zr-Hf-C ternary system. It was based almost exclusively on an extensive 1966 study of phase equilibria [86]. ZrC and HfC form continuous solid solutions with lattice parameter decreasing close to linear from 4.7 to 4.63 for (Zr,Hf)C_0.75_ and linear increase in melting point from 3430 °C ZrC_0.8_ to 3830 °C for HfC_0.98_. Upadhayaya’s cited three sources in Russian from the 1960s, two of which reported a maximum in melting point, and one did not. Complete miscibility between rocksalt HfC and ZrC down to 185 K was predicted from ab initio study using cluster expansion approach [87].

#### Subcarbides

Obata and Nakazawa [88] observed superlattice formation in ZrC_0.7_ (Zr_4_C_3−x_) from X-ray diffraction, resulting in a lattice parameter (9.386 Å) twice of that of rocksalt ZrC. They reported order-disorder transformation to defective rocksalt structure at ~1100 °C. Hu et al. [89] observed domains with Zr_2_C type ordering in ZrC_0.61_ sample prepared by spark plasma sintering (SPS). However, no structures were refined by either author. No Hf subcarbides were reported.

Weinberger and Thompson [68] performed computations on the Zr-C and Hf-C systems using density functional theory and the order-parameter functional method. For the Zr-C system below 1000 °C, they predicted a number of vacancy-ordered structures (Zr_2_C, Zr_3_C_2_, Zr_6_C_5_), which transform to sodium chloride type ZrC_1−x_ at a higher temperature. Their computations indicated that Zr_4_C_3_ phase is unstable, although it is close to ZrC_0.7_ composition in which superlattice reflections were experimentally reported [88]. Subcarbides were not included in the latest Calphad optimizations of the systems. 

### 3.2. Zr-N and Hf-N

A Calphad optimization of Zr-N system was performed by Ma et al. [24] using experimental data from Domagala et al. [90] for Zr-rich part and enthalpies of formation estimated from ab initio computations. The diagram was reassessed by Sridar et al. [91] as part of the modeling of the Ti-Zr-N system using additional thermodynamic data, such as the formation enthalpy of ZrN_1−x_ [92].

The Hf-N system was presented by Okamoto in 1990 [59] from reported experimental data on phase equilibria. No Calphad optimization was performed on a system. We located only one measurement of formation enthalpy of hafnium nitride, which was performed by Humphrey in 1953 [93]. Its reliability is discussed below in a section on rocksalt carbonitrides.

#### 3.2.1. Subnitrides

Subnitrides Hf_3_N_2_ and Hf_4_N_3_ were first prepared by Rudy in 1970 [94] by the reaction of Hf metal with HfN and reported to be stable below 1970 and 2300 °C, respectively. Both compounds have a trigonal structure which can be described as stacking of Hf layers with hexagonal close packing of α-Hf and cubic close packing as in HfN. Rudy [94] refined the structure from powder X-ray diffraction but could not determine if nitrogen atoms are randomly distributed in octahedral interstices or ordered. Lengauer et al. [95] prepared both hafnium subnitride compounds as single phases via diffusion couples and suggested to name them as *eta* (η-Hf_3_N_2−x_) and *zeta* (ζ-Hf_4_N_3−x_) phases to be consistent with known isostructural nitrides and carbides of transitional metal. *Eta* subnitride is observed in the Ti-N system as well. *Zeta* subcarbide phase is known in the Ta-C system and is associated with high fracture toughness. The computational investigation of microstructure and stability of ζ-Hf_4_N_3−x_ together with isostructural Nb and V subcarbides was undertaken by Weinberger et al. (2018) [69]. They concluded that stoichiometric *eta* and *zeta* phases are thermodynamically stable in the Hf-N system, but metastable in carbides. No subnitride phases have been reported for the Zr-N system. 

#### 3.2.2. Higher Nitrides 

In a 1990 review of the Hf-N system, Okamoto stated succinctly that “no information is available on the N-rich side of the diagram” [59]. Ma et al. [57] computed enthalpy of formation for hypothetical ZrN_3_ to be used in Calphad optimizations of the Zr-N system, but no nitrides with N/Me ratio >1 are shown on the final diagram, assessed from 500 to 5500 K. Yet, higher nitrides of Zr and Hf and Ti do exist and are the subject of intense theoretical [96,97,98,99,100,101,102,103,104] and experimental [44,46,97,105,106,107] investigations. 

Zr_3_N_4_ was first synthesized in the 1960s by the reaction of ZrI_4_ [43] and ZrCl_4_ [108] and was shown to be an insulator and to decompose to ZrN and N_2_ above 1100 °C. Twenty years later, it was rediscovered by Schwartz et al. [46] at the IBM research center, who obtained transparent films while varying the N/Zr ratio in the dual ion-beam sputtering process. It took 10 more years before Lerch et al. [44] refined the Zr_3_N_4_ crystal structure from powder sample as orthorhombic (*Pnma*). The isostructural Hf_3_N_4_ was not reported. The hardness of orthorhombic Zr_3_N_4_ is similar to that of rocksalt ZrN. The transition from transparent insulator to metallic coating is of independent interest for potential applications.

In 2003, the synthesis of cubic Zr and Hf higher nitrides with Th_3_P_4_ (***I*-**43***d***) structure was reported in laser-heated diamond cell experiments at 18 GPa at ~2500 °C [45] and, two years later, Zr_3_N_4_ was synthesized in this phase at atmospheric pressure by reactive evaporation technique [109]. The hardness of the c-Zr_3_N_4_ coating exceeds those of ZrN and TiN.

Recent ab initio computations showed that orthorhombic (*Pnma*) Hf_3_N_4_ is stable at ambient pressure [110,111]. However, Zhang et al. [103] found using first principles evolutionary calculations that *Pnma*-Hf_3_N_4_ is stable at 2–9 GPa range (while a Zr_3_N_4_ analog was synthesized [44] at ambient pressure) but predicted that triclinic and monoclinic higher nitrides Hf_11_N_12_, Hf_7_N_8_, and Hf_4_N_5_ and Hf_3_N_4_ are stable at zero pressure (Table 1).

### 3.3. Zr Versus Hf in Binaries with Carbon and Nitrogen 

Zirconium and hafnium form rocksalt monocarbides and mononitrides with full mutual solubility, at least above 1000 °C. However, substantial differences are observed between the Zr and Hf systems with carbon and nitrogen.

Zr analogs of *eta* (η-Hf_3_N_2_) and *zeta* (ζ-Hf_4_N_3_) hafnium subnitrides were not reported (Table 1), neither was synthesis of an Hf analog of Zr_3_N_4_ at ambient pressure. In metal-rich compositions, hafnium dissolves more carbon and nitrogen than zirconium, which results in stabilization of α-Hf to higher temperatures. In contrast with Zr, β-Hf is not stable in the presence of HfC. A more detailed study of N positions in α-Hf may very well uncover different structures in what is now labeled as the α-Hf(N) solid solution field (Figure 2). 

It must be noted that assessments of Zr and Hf binaries with carbon and nitrogen are based on experimental data obtained mostly in the 1960s, and authors are often forced to choose between conflicting datasets. For example, Bitterman and Rogl’s (1997) assessment of Hf-C diagram follows the experimental diagram by Rudy (1965). Upadhayaya (1996) favors the diagram by Sara [79], which shows that the α-Hf phase dissolves up to 18 at.% of C and is stable to 2820 °C. The impurities of O and N in carbide samples used in different studies could shift the observed equilibrium drastically. Thus, we expect that phase relations in the binary systems of Hf and Zr with C and N may undergo substantial revisions and include higher nitrides, subnitrides, and subcarbides as new experimental and computational data become available. 

### 3.4. Zr-C-N, Hf-C-N, Zr-N-O, Hf-N-O Ternaries

The available data on phase equilibria and thermodynamics of ternary systems are scarce and not systematic. Lerch [118] described several ternary compounds in the ZrO_2_-Zr_3_N_4_ pseudobinary, which include Zr_2_ON_2_ (γ-phase with bixbyite structure) and several other fluorite-related structures denoted as β, β’ and β’’ and listed in Table 1. Above 1000 °C all of them decompose to NaCl-type ZrN and N-stabilized fluorite-type zirconia. Most of the experimental and computational studies were devoted to rocksalt carbonitrides thermochemistry and properties. These efforts are reviewed in the next section.

## 4. Rocksalt (oxy)carbonitrides

The rest of the review is focused on structure and thermodynamic properties of (oxy)carbides and (oxy)nitrides of Zr and Hf. Among them, much more data are available for ZrC and were summarized, analyzed, and discussed in an excellent review by Jackson and Lee [81]. The papers by Lengauer et al. [3,38,39,40,41,112,124] provide analysis of solid state properties of Zr and Hf compounds in the larger frame of transition metal carbonitrides.

Our aim here is to aid future experimental work, ab initio computations and Calphad optimizations by bringing to light to some relevant data which are often omitted, e.g., formation enthalpies for oxycarbides measured by Kornilov [125], work of Turchanin [30] on compositional dependence of high temperature heat capacity of carbonitrides, and Brundiers [126] work on Hf oxycarbonitrides. We also want to highlight very recent developments, such as the application of the impulse current heating method to measure high-temperature heat capacities and fusion enthalpies for zirconium carbonitrides and ab initio computations of the fusion enthalpies and melting temperatures in the Hf-C-N system. 

### 4.1. Structural Features 

Ti, Zr, and Hf carbides and nitrides are found in NaCl-type (rocksalt) structure. Only Ti forms an NaCl-type metallic oxide, with stoichiometry varying from TiO_0.75_ to TiO_1.3_ [127]. Besides the alkali halides, the oxides of alkaline earths, many divalent transitional metals (Mn, Fe, Ni) and vanadium monoxide all share a NaCl structure. All rare earth elements (REE) form NaCl-type nitrides, oxycarbides, and carbonitrides but not stoichiometric monocarbides [128]. The monoxides of La, Ce, Nd, Sm, Eu, Y, and Yb with rock salt structures were reported in the 1950s and 1960s [129]. They were synthesized by reduction of sesquioxides with carbon or a corresponding rare earth metal. However, it was later established [130,131] that only divalent YbO and EuO can be synthesized at ambient pressure and earlier reported monoxides of other rare earth elements were, in fact, oxycarbides or oxynitrides (although metallic oxides of trivalent light REE were synthesized in the rocksalt structure at 1.5–8 GPa and 800–1000 °C) [132]. Thorium and uranium also form NaCl-type nitrides and carbides [133], and their potential application as advanced nuclear fuels with high loading is the reason for the keen interest in ZrC as a protective coating and in rare earth carbides as fission products.

**Figure 3:**
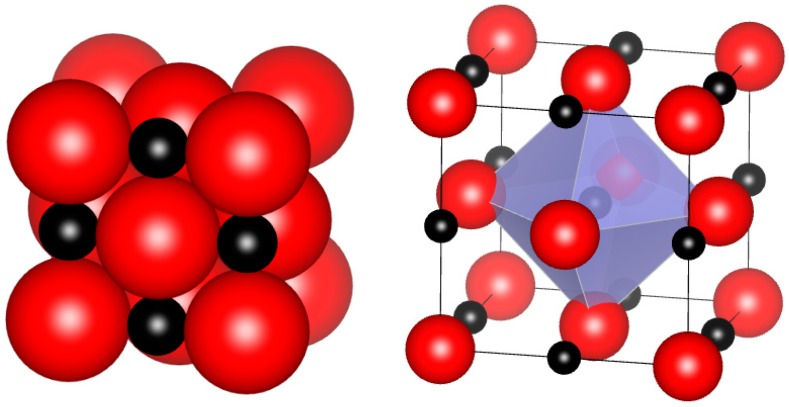
Rocksalt structure in space-filling and polyhedral representations, showing the location of nonmetal atoms in octahedral voids of a face-centered cubic metal sublattice. Red spheres indicate metal atoms.

The rocksalt structure (Figure 3) can be des**c**ribed as two face-centered cubic lattices of metal and nonmetal moved with respect to each other or as a metal cubic close-packed structure with all octahedral voids occupied by nonmetal. The space group is Fm3m, with coordinates for all atoms defined by space group symmetry: the metal at 0, 0, 0 and the nonmetal at ½, ½, ½. Four formula units make up the conventional cubic unit cell (Z = 4). The structure can accommodate vacancies on both metal and non-metal sublattices. The carbon-to-metal ratio in Zr and Hf carbides is always less than or equal to 1; thus, their formula is often written as MeC_1−x_. This formula does not mean that there are no vacancies on the metal sublattice; it only indicates that there are typically fewer vacancies on metal sublattice than on the carbon sublattice. A notable exception is the case of nitrides, since compositions with nitrogen excess, up to HfN_1.2_ have been reported in the rocksalt structure [126]. The formula could equivalently be written as Hf_1−x_N, to indicate that the vacancies are predominantly on the metal sublattice.

**Figure 4:**
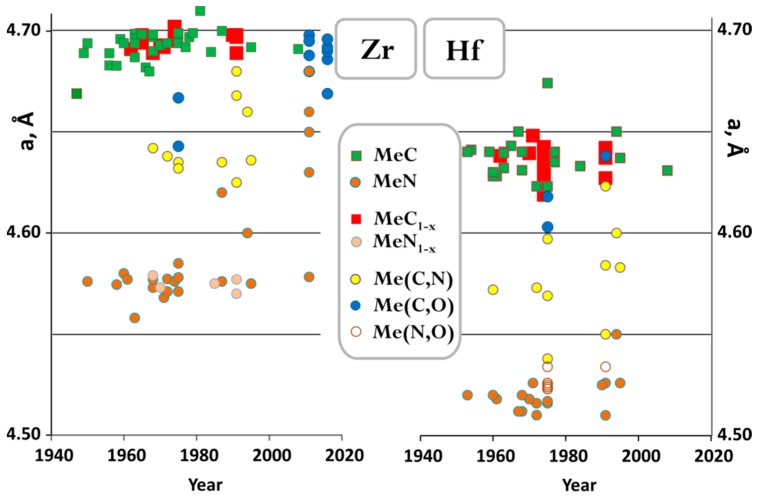
The lattice parameter of NaCl-type compounds. (**Left**) Hf-C-N-O system. (**Right**) Zr-C-N-O system. See Table S2 for compositions for each data point.

Figure 4 shows reported lattice cell parameters for Zr and Hf carbides nitrides and their solid solutions. The considerable variation in cell parameters for nominally ZrN composition reported in recent years is likely related to the fact that the thin films samples were produced by CVD and PVD. Due to analytical difficulties in quantifying ratios of C, N, and O in nonstoichiometric carbonitrides, it is tempting to establish lattice parameter composition relations for (oxy)carbonitrides, and many analytical expressions were derived and reported in separate studies [55,81,113,114,134]. However, these parametrizations are likely to be useful only within a set of samples arising from the same synthesis procedure. Cell parameter for Zr and Hf carbides can be lowered by either carbon deficiency, or by O or N substitution. To complicate things even further, the lattice parameter of ZrC_x_ as a function of C content shows a maximum at x = 0.8, while this is not observed for HfC_x_ (Figure S1). Hydrogen can also be incorporated in the vacancies of the nonmetal sublattice without a significant effect on the lattice parameter [35]. In this respect, the few reports on the formation enthalpies of these compounds discussed below are valuable references, since in order to derive formation enthalpies from heats of combustion, initial samples, and combustion products were thoroughly characterized, often using a combination of different techniques.

There are no experimental reports on the high pressure phase transformations of rocksalt-type carbonitrides. Ab initio computations showed that the NaCl structure is stable for ZrC, ZrN, and HfN to at least 150 GPa. The predicted high pressure phase of carbonitride is a CsCl-type structure [67,110], in which coordination increases from 6 to 8 with an associated volume decrease of less than 1.4 %. An unexpected computational result from Zhang et al. [103] is a prediction that HfN is thermodynamically stable in the rocksalt structure only above 60 GPa or above 400 °C at zero pressure and the ground state of HfN is a hexagonal P63/mmc (TiAs-type) structure, which was never reported in experiments. 

### 4.2. Stability Field of Rocksalt Carbonitrides in Hf-Zr-C-N-O System

Most experimental studies of compositions of rocksalt carbonitrides have been performed on the samples synthesized at 1000–2500 °C. At these temperatures, complete miscibility in the rocksalt structure is observed between ZrN-ZrC and HfN-HfC [112,124] (and presumably in ZrC-ZrN-HfN-HfC). The main questions remaining pertain to the magnitudes of the thermodynamically stable stoichiometry deviations due to deficiencies on either metal or nonmetal sublattices and to oxygen and hydrogen dissolution in the structure. 

Binder et al. [112] reported that the maximum deficiency on non-metal sublattice for zirconium carbonitrides at 1150 °C is nearly constant between ZrN_1−x_ and ZrC_1−x_ and is limited to ~60 at% Zr. Brundiers [126] reported isothermal crosssection for Hf-C-N system at 1500 °C showing close to linear Hf-rich boundary from HfC_0.6_ to HfN_0.7_. He also studied carbon- and nitrogen-rich compositions and found that deficiency on the Hf sublattice does not occur in solid solutions with more than 20 % HfC (Figure S2).

Constant et al. [114,135] studied oxygen solubility in zirconium and hafnium carbonitrides and their solid solutions. They prepared samples from metallic Zr and Hf mixed with carbides, nitrides, oxides, and carbon. They reported the stability fields in the form of pseudo-ternary crosssections MeO-MeC-MeN (with Me = Zr or Hf). This choice of the end members assumes no deficiency on either the metal or nonmetal sublattices. They found extensive oxygen solubility in zirconium carbonitrides with x in ZrC_1−x_O_x_ increasing from 0.55 at 1600 °C to 0.75 at 2000 °C, and from 0.35 to 0.55 in ZrN_1−x_O_x_. They reported that Hf(N,C)_1−x_O_x_ compositions have lower oxygen solubility limits with maximum values extending from x = 0.2–0.25 at 1600 °C to x = 0.25–0.3 at 2000 °C. 

A new investigation of oxygen solubility in ZrC and HfC was published by Rejasse et al. in 2016 [55] and 2017 [113]. They confirmed the higher solubility of oxygen in ZrC relative to HfC but reported much lower limits: x = 0.26 for ZrC_1−x_O_x_ at 1850 °C and x = 0.10 for HfC_1−x_O_x_ at 1750 °C. Kornilov et al. [136] performed a characterization of Hf oxycarbides (Table S3) for the purpose of formation enthalpy determination and reported a maximum oxygen content of x = 0.15 in the substoichiometric HfC_0.6_O_0.15_ composition. 

Note that in Rejasse’s [113] experiments, the samples were synthesized by a carbothermic reduction of oxides with amorphous carbon, which rules out deficiency on the non-metal sublattice. Constant [114,135] and Kornilov [136] both reported higher oxygen dissolution limits in Zr and Hf rocksalt oxycarbides. They used metallic Zr and Hf in the synthesis, thus making metal-rich compositions possible. 

The quantitative analysis of oxygen in oxycarbides is experimentally challenging and can be a major source of discrepancies. Rejasse [113] derived free carbon and total carbon from an analysis of CO_2_ evolved on combustion in oxygen at 1000 °C and 1800 °C, respectively, and oxygen was analyzed from CO evolved on samples during annealing at 2500–2800 °C in He atmosphere and graphite crucibles. This procedure assumes that above 2500 °C no oxygen is retained in carbonitrides. Kornilov [136] determined oxygen content by both vacuum fusion method and neutron activation analysis and reported that the former substantially underestimate oxygen content, apparently due to irreversible sorption of combustion products. Recently, ion microprobe methods were also applied for oxygen analysis in carbonitrides [137]. 

### 4.3. Melting Temperatures of Hafnium Carbides and Carbonitrides

Due to similar chemistry, separating hafnium and zirconium have historically been relatively difficult. The element hafnium was not identified until 1923, making it the last stable element to be discovered. Early studies of the Hf-C system [21,138,139,140,141,142] often involved an impurity of several wt% of zirconium. Given that ZrC melting temperature is 400 °C lower than HfC (Table 2), this may affect early results in the Hf-C system.

The melting temperature of HfC was first determined as 4160 K (3887 °C) by Agte and Alterthum in 1930 [138]. They also performed melting temperature measurements of binary carbides of Zr, Nb, Mo, Hf, Ta, and W their ternary solid solutions and found that rocksalt compound with composition Ta_4_HfC_5_ had a melting temperature measured as 4215 K (3942 °C). Although not confirmed by later measurements by Rudy et al. [142], Ta_4_HfC_5_ had often been considered as the material with the highest known melting point.

The interest in HfC increased significantly in the space age of the 1960s when there was an urgent need for materials with high melting temperatures, high softening temperatures, and other high-temperature properties so that these materials would stand up under extreme temperature conditions, such as in rocket engines or during atmospheric reentry. In 1963, Admas and Beall [140] determined that the highest melting point in the Hf-C system is 3895 °C at HfC_0.985_. In 1965, Sara [141] investigated the Hf-C phase diagram by metallography and X-ray diffraction. The most refractory composition was established at HfC_0.90_, with a melting temperature of 3830 °C. In 1967 Rudy and Progulski [143] determined the melting temperature to be 3928 ± 40 °C at HfC_0.942_. In the same year, Deardorff et al. [144] found that hafnium carbide with stoichiometry from HfC_0.96_ to HfC melts at ~3840 °C corroborating Sara’s results [141], and Storms [21] reported HfC melting temperature at 3950 °C which is within the range of Rudy’s measurements [143].

In 2016, new measurements of the melting temperature of hafnium carbide were performed, for the first time in the last fifty years. From laser melting experiments, Cedillos-Barraza et al. [145] reported the value 4232 ± 84 K (3959 °C) for HfC_0.98_ composition. The latest experimental measurement was performed by Sheindlin et al. (2018) [62] (also by laser melting) and yielded similar value (3982 ±30 °C) for close to stoichiometric HfC. These latest determinations overlap within reported uncertainties with 1967 measurements by Rudy and Progulski (3928 ± 40 °C) performed by Joule heating with Pirani furnace [143].

A computational study of high temperature thermodynamics of Hf-C, Hf-C-N, and Hf-Ta-C systems was reported by Hong and van de Walle in 2015 [1] using density functional theory (DFT) [146]. While the computational methods used are found to reliably predict trends in melting points as a function of composition, obtaining absolute melting points is more challenging. To assess the sensitivity of the results to the choice of exchange-correlation functional, they considered both the standard Perdew-Burke-Ernzerhof (PBE) functional [147] and the more accurate (but more computationally expensive) hybrid Heyd-Scuseria-Ernzerhof (HSE) functional [148], the latter being implemented perturbatively, for computational efficiency purposes. The highest melting point located at the HfC_0.81_ composition was found to be of 3962 K (3689 °C) using PBE and 4422 K (4149 °C) using HSE. The true melting point should be closer to the HSE value but the difference in the two values is an indication of the order of magnitude of accuracy. Previous experimental values are indeed found to lie within the range spanned by the PBE and the HSE values. 

**Figure 5:**
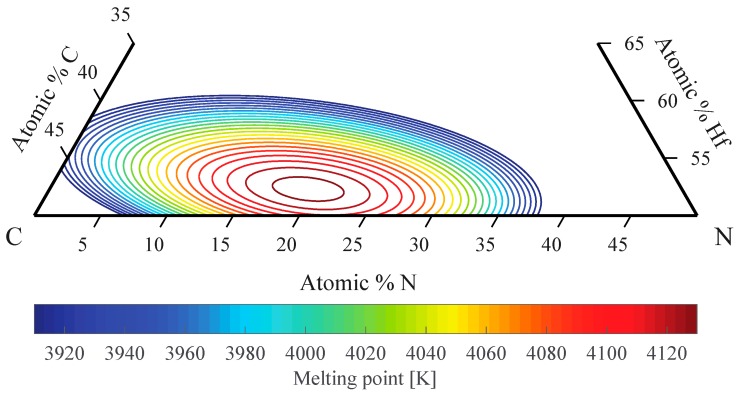
Melting temperatures of Hf-C-N alloys, based on density functional theory (DFT) calculations. Melting temperature maximum is located at Hf_0.53_C_0.27_N_0.20._ Modified after Hong and van de Walle [1].

In the Hf-Ta-C system, PBE values for melting temperature of Ta_4_HfC_5_ and HfC were found to be within computational uncertainties; however, in the Hf-C-N system, the pronounced maximum in melting temperature was discovered [1]. The highest melting point was predicted to be 4126 K (3853 °C) at the composition Hf_0.53_C_0.27_N_0.20_, based on the PBE functional [1]. Compared to the PBE melting point of 3962 K in the Hf-C system, the Hf-C-N system further increases the melting point by 160 K (Figure 5). As predicted composition trends are more reliable than absolute numbers, these results suggest that the true melting point of Hf_0.53_C_0.27_N_0.20_ is best estimated by adding 160 K to known experimental result for the Hf-C system. Taking experimental melting temperature for HfC as 3888–4012 °C (lower and upper limits from Rudy and Progulski [143] and Sheindlin et al. [62]), the melting temperature at Hf_0.53_C_0.27_N_0.20_ is thus estimated at 4048–4172 °C which makes it a material with highest known melting point. 

### 4.4. Thermochemistry 

Standard enthalpies of formation, entropies and heat capacities for rocksalt carbides and nitrides of Zr and Hf are listed in Table 1. The values for Ti compounds are also shown for comparison. 

#### 4.4.1. Enthalpies of Formation

**Figure 6:**
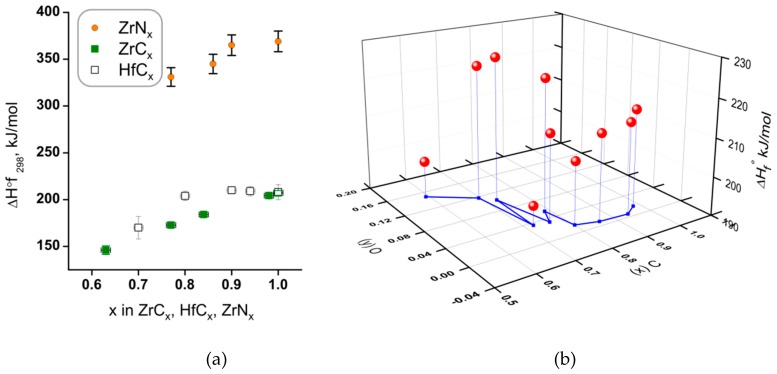
(**a**) Standard enthalpies of formation of Zr and Hf carbides and Zr nitrides as a function of carbon content. (**b**) Standard enthalpies of formation of 10 hafnium oxycarbides HfC_x_O_y_ plotted measured by Kornilov et al. [125], from which the values for HfC_x_ were derived. Note that the oxygen content reaches y = 0.15 for most carbon deficient composition. See Tables S3 and S4 for reported uncertainties.

Enthalpies of formation of Zr and Hf carbides and Zr nitride were determined by several groups using combustion calorimetry in oxygen bomb. Due to differences in reported values for Zr carbides, Kornilov et al. in 1975 [136] performed a new experimental study on seven samples of ZrC_0.72_ to ZrC_0.99_ (Figure 6) and found close agreement with the 1969 work by Baker, Storms and Holley [149]. The same group reported new measurements on the formation enthalpy of hafnium carbide in 1977 [125]. 

The value of the formation enthalpy of ZrN included in a Glushko’s reference book (371.5 ± 1.2 kJ/mol is consistent with the latest measurements performed by Galbraikh et al. [92] for stoichiometry ranging from ZrN_0.77_ to ZrN. They have also measured the formation enthalpy for one oxynitride (see Tables S2 and S3 in supporting information for all compositions). In the latest optimization for Zr-N system, Sridar et al. [91] tabulate all the experimental values, varying from −336 to −365 kJ/mol. They also obtained the new value −341 kJ/mol by ab initio methods and arrived at a value of −350 kJ/mol based on a Calphad optimization.

There is a single measurement of formation enthalpy of HfN reported by Humphrey in 1953 [93]. He reports ΔHf_298_ as 369.2 ± 1.4 kJ/mol from eight measurements of heats of combustion, using ~5 g of HfN in each experiment. Samples were prepared by nitridation of metallic Hf at 1400–1500 °C; the lattice parameter was not measured, and a HfN stoichiometry was confirmed from weight gains on nitridation and combustion. In the same work, Humphrey also reported measurements of the formation enthalpy of HfO_2_, which is within 5 kJ/mol from currently accepted value [154]. Thus, we conclude that despite the fact that no new measurements on HfN were made for 60 years; there is no reason to suspect gross errors in HfN formation enthalpy reported by Humphrey [93]. No value for HfN formation enthalpy is included in the NIST-JANAF Thermochemical Tables [152]. The value for HfN given in Glushko’s Thermal Constants of Substances [150] is −373.6 kJ/mol, and apparently includes minor adjustment in treatment of original data by Humphrey [93], which is the only source cited.

A systematic error is likely affecting data on formation enthalpies from combustion calorimetry on Hf and Zr metals, carbides, and nitrides. This should be kept in mind when comparing experimental data with ab initio calculations [155]. It comes from neglecting the contribution of surface energy of fine grained (nanophase) monoclinic HfO_2_ and ZrO_2_ formed as combustion products. The value for surface energy of monoclinic HfO_2_ was unknown at the time of combustion calorimetry experiments. However, recent experimental results found the value 3.7 J/m^2^ and accounting for it can change formation enthalpy measured by combustion experiments by as much as −20 kJ/mol [156]. 

#### 4.4.2. High Temperature Heat Capacities from Calorimetry 

**Figure 7:**
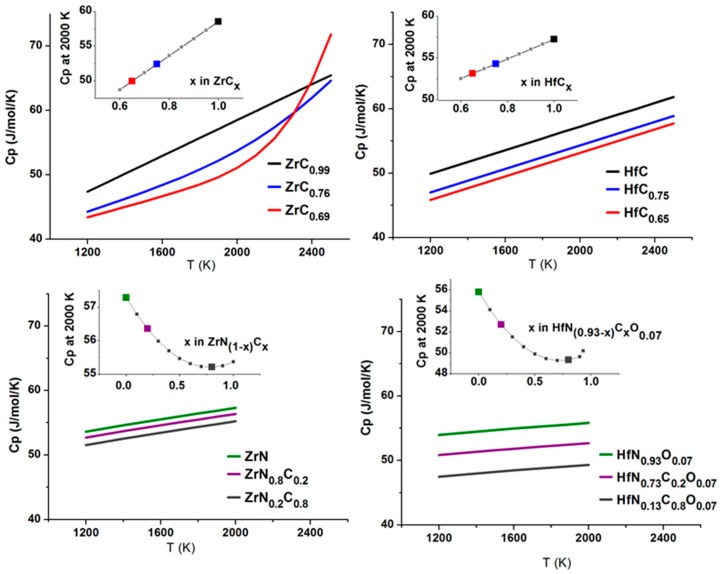
High temperature heat capacity (Cp) of zirconium and hafnium carbides and carbonitrides after Turchanin (1991) [30]. The insets show isotherms of heat capacity at 2000 K versus composition. Temperature dependence for selected compositions is shown on the main graphs. The data are tabulated in supporting information (Tables S6 and S7).

Ti, Zr, and Hf nitrides have slightly higher heat capacities than carbides (Table 2). The evaluations for ZrC and ZrN in NIST-JANAF Thermochemical tables [152] are of 1964 vintage with no data for Hf analogs. The later evaluation by Storms [21] for ZrC_0.96_ and HfC_0.98_ are well known [22,81]. Turchanin et al. [30,31,32] measured high temperature enthalpies for Zr, and Hf carbonitrides by drop calorimetry and derived heat capacities with an estimated uncertainty of 1.5 %. His results are shown in Figure 7 and summarized below.

Turchanin’s measurements were performed on samples 3–7 g in weight under high vacuum (~6·10^−6^ Torr). The enthalpy increments were measured to 2227 °C (2500 K) for ZrC_x_ and HfC_x_ and to 1727 °C (2000 K) for ZrN_1−x_C_x_ and HfN_1−x_C_x_. All hafnium carbonitride samples studied by Turchanin were prepared from a mixture of carbides and nitrides at 2200–2800 °C and contained some oxygen and tabulated data are related to oxycarbonitrides with composition HfN_(0.93−x)_C_x_O_0.07_. Heat capacity per mole of ZrC_x_ and HfC_x_ increases linearly with x, as expected due to the increasing total number of atoms per mole of compound. For carbon deficient zirconium carbides, Turchanin found that the Cp increase with temperature deviates from a linear behavior above ~1800 °C. This effect is likely related to the thermally activated motion of carbon atoms, so-called “sublattice melting” [157,158]. Turchanin did not observe this change in the rate of heat capacity increase for defective hafnium carbide. However, that does not indicate that this effect is absent in HfC_1−x_; its melting temperature is ~400 °C higher than for zirconium carbide and the effect may not appear in the lower temperature range of the measurements (below 2227 °C).

For the carbonitrides, ZrN_1−x_C_x_ and HfN_1−x_C_x_, the heat capacity decreases with carbon content, but not linearly, with a minimum in Cp for a composition of x = 0.8. Turchanin notes that this minimum corresponds to the composition with the strongest bonding since, in carbonitrides, the electronic contribution to heat capacity does not change substantially.

Lengauer et al. [40] studied the heat capacities of several Hf and Zr carbonitride compositions by differential scanning calorimetry (DSC) below 1000 °C and reported good agreement with the corresponding Turchanin values for Zr carbonitrides, althought the limited number of compositions in Lengauer’s study did not allow him to confirm the minimum in Cp reported by Turchanin. Recently, Ciriello et al. [6] performed measurements of the heat capacity of pure ZrN from 1.8 K, as part of a study of the thermal conductivity of (ZrPu)N solid solutions. Their Cp value for ZrN at 1200 °C (52 J/mol/K) is in good agreement with Turchanin’s evaluation (Table 2).

#### 4.4.3. Fusion Enthalpies of ZrC and ZrN from Pulsed Heating

The method of pulsed heating [74,159,160] originated from the “exploding wire” technique [160] and was extremely useful for the determination of high temperature resistivities, heat capacities, and fusion enthalpies of metals. It relies on the pyrometric measurement of sample temperature increase on capacitors discharges through the metal wire. Due to the microseconds timeframe of discharge, the heat losses can be neglected, and the total energy is known from the capacitor calibration. Recently, this approach was successfully applied for carbides and nitrides films 100 µm or thinner [61,63,64,65,66,161,162] and the first experimental data on fusion enthalpies and high temperature heat capacities of ZrC and ZrN were reported. The fusion enthalpy of ZrN was measured as 104 kJ/mol [65], and the fusion enthalpy of ZrC_x_ was found to increase from 92 kJ/mol for ZrC to 111 kJ/mol for ZrC_0.95_. The experimental setup described by Savvatimskiy et al. [63] provides black body-like geometry for measurements by a sighting pyrometer inside the wedge formed by glass plates coated with the sample. We expect that future applications of the “exploding wire” technique to metallic nitrides and carbides will result in an explosion in much needed thermodynamic data for Calphad modeling of carbonitride systems and for testing the predictions of ab initio computations.

#### 4.4.4. Fusion Enthalpies of HfC-HfN from ab Initio Computations

Based on DFT calculations by Hong and van de Walle (2015) [1], HfC_x_ has an exceptionally large fusion enthalpy, as high as 0.81 eV/atom. This is a value unparalleled among refractories. (For reference, Al_2_O_3_ (m.p. 2345 K): 0.22; W (m.p. 3695 K): 0.37; Hf (m.p. 2506 K): 0.26 eV/atom). Estimated fusion enthalpies of HfC_x_ and HfC_x_N_y_ are summarized in Table 3. Note that these fusion enthalpies were obtained as a byproduct in the process of melting temperature calculation [1], using the small-size coexistence method [163]. As a result, they were slightly underestimated, due to high defect concentration in the freshly formed solid phase. A more accurate method, which models the solid and liquid phases in separate simulations, typically increases the fusion enthalpy by 0.05–0.10 eV/atom in these systems.

While there is no experimental fusion enthalpy of HfC_x_ for comparison, the computational value, 0.81 eV/atom or 147 kJ/mol, is consistent with the experimental values of the fusion enthalpy of ZrC_x_ from pulse heating experiments reviewed above, given that the melting temperature of ZrC_x_ (3572 °C) is lower than that of HfC_x_ (3982 °C) by 10%.

## 5. Summary and Future Directions

Despite the sharp increase in the number of publications on carbides, the literature’s focus has been on either widely used Ti carbonitrides, or on multicomponent (so-called high entropy) compositions, and large areas remain to be mapped computationally and experimentally. For instance, no thermodynamic optimization for the Hf-N system has been performed. Only isothermal crosssections were assessed for the Hf-C-O and Zr-C-O and Hf-C-N and Zr-C-N systems, and there are conflicting experimental results on oxygen solubility in HfC and ZrC. This points to a critical need for fundamental data to be further integrated into suitable thermodynamic models. Oxidation behavior and interaction with water vapor is of paramount importance for the use of carbides in high temperature flight and fission reactors related applications, yet limits of oxygen and hydrogen solubility in Zr and Hf carbides and nitrides are not reliably established as a function of temperature.

Prompted by the recent prediction of highest melting temperature in Hf-C-N system [1] we screened the literature for possible experimental evidence and found two reports indicating that some compositions of Hf(C,N) solid solution might indeed have higher bonding strength than end members: (i) Brundiers in 1975 [126] reported that the HfC_0.58_N_0.42_ composition had the highest hardness and smallest thermal expansion in the Hf-C-N system; (ii) Turchanin in 1991 [30] reported minima in heat capacities isotherms for Hf(C,N) and Zr(C,N) compositions. Since the electronic contribution to the heat capacity should not vary much from carbides to nitrides, the difference is likely coming primarily from lattice vibrations and compositions with the heat capacity minima are associated with higher-frequency phonon spectra and thus the strongest bonds [164].

Brunder’s [126] work is a PhD thesis published in German, and Turchanin’s predictions [30] were published in a monograph in Russian. It is thus no surprise that they mostly escaped attention until now. Still, a direct experimental confirmation of the high melting points predicted in the Hf-C-N system [1] would be welcome. In addition, as high melting temperature correlates with high hardness, a study of Zr and Hf carbonitrides may provide coatings superior to Ti carbonitrides currently used.

## Figures and Tables

**Table 1:** Phases reported and predicted in Hf-C-N-O and Zr-C-N-O systems at ambient pressure.

Phases	SG	Str. Type	Comments, [Refs]
**Reported from the experiments at 1 Atm**			
δ-Hf,Zr(C,N,O)	*Fm*-3*m*	NaCl (B1)	[38,55,112,113,114]
η-Hf_3_N_2_	*R*-3*m*	Ta_2_VC_2_	[94,95]
ζ-Hf_4_N_3_	*R*-3*m*	Hf_4_N_3_	[94,95]
Zr_3_N_4_	*Pnma*		[115]
Zr_3_N_4_	*I*-43*d*	Th_3_P_4_	[109]
β-Zr_7_O_8_N_4_	R-3*H*	Pr_7_O_12_	[116]
β’-Zr_7_O_11_N_2_	*R*-3*H*	Zr_5_Sc_2_O_13_	[117]
β”-Zr_7_O_9.5_N_3_			[118]
γ-Zr_2_ON_2_, Hf_2_ON_2_	*Ia*-3	Mn_2_O_3_	[119]
Zr_4_O_5_N_2_	*I*4*cm*	Flr-deriv	[120]
**Predicted from computations to be stable at 1 atm**			
Zr_3_C, Hf_3_C,	*Pnma*	In_3_Ir	[121]
Zr_3_C_2_ Zr_8_C_7_			[122]
(Zr,Hf)_2_ON_2_	*C*1*m*1	Pv-deriv	[123]
Hf_6_N	*P*-31*c*		[103]
Hf_2_N	*Pnnm*		[103]
HfN (ZrN)	*P*63/*mmc*	TiAs	[103]

**Table 2:** Selected thermodynamic properties for rocksalt carbides and nitrides of Ti, Zr, and Hf: lattice parameters, enthalpies of formation, bond dissociation energies, standard entropies, heat capacities, melting temperatures, and thermal expansion coefficients.

Lattice parameter a, Å [35]		ΔH°f _298_ kJ/mol [150]	D_0_ kJ/mol [150]	S_298_ J/mol/K [150]	Cp_298_ J/mol/K [150]	Cp_2000_ J/mol/K [30]	Tm, °C	α 10^−6^/K [23]
TiC	4.33	−209 ± 21 §	1388 ± 20	24.7 ± 0.2	34.3 ± 0.3	60.5	3067 ± 25 [143]	7.4
TiN	4.24	−338 ± 4	1261	30.3 ± 0.2	37.1 ± 0.1	61.2	2945 ± 30 [151]	9.3
ZrC	4.70	−207 ± 3	1508 ± 7	33.3 ± 0.1	37.9 ± 0.8	55.4 ǁ	3572 ± 30 [62]	6.7
ZrN	4.57	−372 ± 2 †	1438 ± 6	38.9 ± 0.2	40.4 ± 0.1	57.3	2955 ± 30 [151]	7.2
HfC	4.64	−208 ± 8	1537 ± 9	40.1 ± 0.2	38.1 ± 0.2	50.2§	3982 ± 30 [62]	6.6
HfN	4.52	−374 ± 2 ‡	1461 ± 5	45 ± 1	41 ± 2	55.8 *	3330 ± 50 [151] §	6.9

ǁ cf. Cp_2000_ 56.3 J/mol/K for ZrC_0.96_ from Storms [21,22] and 57.4 J/mol/K for ZrC from NIST-JANAF [152]; § cf. ΔHf_298_ (TiC) −186 ±18 from Meschel and Kleppa (2001) [153]; † cf. ΔHf_298_ (ZrN) −350 kJ/mol from Calphad optimization [91]; ‡ cf. ΔHf_298_ (HfN) 369.2 ±1.4 kJ/mol from original work [93]; § refers to HfC_0.93_O_0.07_, cf. Cp_2000_ (HfC_0.98_) 57.1 J/mol/K from Storms [21,22]; * The value refers to HfN_0.93_O_0.07_ [30]; § Tm (HfN) was reported to increase with N pressure from 2920 °C at 0.01 atm to 3810 °C at 80 atm [59].

**Table 3:** Fusion enthalpies of HfC_x_ and HfC_x_N_y_ from DFT calculations [1].

HfC_x_	Fusion Enthalpy		HfC_x_N_y_	Fusion enthalpy	
	(eV/atom)	(kJ/mol)		(eV/atom)	(kJ/mol)
HfC	0.67	130	HfC_0.75_N_0.22_	0.79	150
HfC_0.97_	0.68	130	HfC_0.62_N_0.19_	0.71	124
HfC_0.94_	0.76	141	HfC_0.56_N_0.25_	0.73	127
HfC_0.91_	0.72	133	HfC_0.56_N_0.38_	0.75	141
HfC_0.88_	0.73	131	HfC_0.44_N_0.5_	0.74	139
HfC_0.84_	0.73	130	HfC_0.31_N_0.62_	0.69	130
HfC_0.81_	0.72	126			
HfC_0.78_	0.69	118			
HfC_0.75_	0.69	117			

## Supplementary Materials

The following are available online at https://www.mdpi.com/1996-1944/12/17/2728/s1, Table S1: The selected reactions for preparation of carbides and nitrides of Zr and Hf; Table S2: Unit cell parameters for NaCl–type compounds reported in Hf-Zr-C-O-N system; Figure S1: Dependence of unit cell parameters of substoichiometric Zr and Hf carbides from carbon content; Table S3: Standard enthalpies of formation of HfC_x_O_y_ compounds; Table S4: Standard enthalpies of formation of ZrN_x_O_y_ compounds; Table S5: Heat capacities of Zr and Hf carbides; Table S6: Heat capacities of Zr carbonitrides; Table S7: Heat capacities of Hf carbonitrides; Figure S2: Compositions of NaCl-type hafnium carbonitrides in Hf-C-N system at 1500 °C and 1 atm N_2._
