# Supplementary material for: Carbides and Nitrides of Zirconium and Hafnium
Source: Materials (Basel). 2019 Aug 26;12(17):2728. doi: 10.3390/ma12172728 (PMC6747801; doi:10.3390/ma12172728)
Supplement: Supplementary file 1 [file materials-12-02728-s001.pdf]

materials

*Supplementary materials for the manuscript*

# Carbides and Nitrides of Zirconium and Hafnium

**Sergey V. Ushakov [1,*], Alexandra Navrotsky [1,*], Qi-Jun Hong [2,*] and Axel van de Walle [2,*]**

[1] Peter A. Rock Thermochemistry Laboratory and NEAT ORU, University of California at Davis, Davis, CA 95616, USA
[2] School of Engineering, Brown University, Providence, RI, 02912, USA
* Correspondence: svushakov@ucdavis.edu (S.V.U.); anavrotsky@ucdavis.edu (A.N.); qhong@alumni.caltech.edu (Q.J.H.); avdw@brown.edu (A.W.)

**Table S1.** The selected reactions for preparation of carbides and nitrides of Zr and Hf.

| Reaction | $\Delta H_{rxn}$ kJ/mol | $\Delta S_{rxn}$ J/mol/K | Note‡ |
|---|---|---|---|
| **Carbides preparation** | Graphite reaction with metal or hydride | | |
| Zr(s) + C (graphite)= ZrC(s) | -196 | -11 | |
| Hf(s) + C (graphite)= HfC(s) | -230 | -8 | |
| $ZrH_2$(s) + C (graphite)= ZrC(s) +$H_2$(g) | -276 | 123 | |
| $HfH_2$(s) + C (graphite)= HfC(s) +$H_2$(g) | NA | NA | |
| | Carburizing gas reaction with metal | | |
| Zr(s) + 2CO(g) = ZrC(s) + $CO_2$(g) | -317 | -191 | ΔG < 0 at T < 1750 °C |
| Hf(s) + 2CO(g) = HfC(s) + $CO_2$(g) | -361 | -201 | ΔG < 0 at T <1950 °C |
| | Carbothermal process | | |
| $ZrO_2$(s) +3C (graphite) = ZrC(s) + 2CO(g) | 680 | 361 | ΔG < 0 at T > 1652 °C |
| $HfO_2$(s) + 3C (graphite) = HfC(s) + 2CO(g) | 662 | 360 | ΔG < 0 at T > 1642 °C |
| **Nitrides preparation** | Metals/metal hydrides nitridation | | |
| Hf(s) + ½ $N_2$(g) = HfN (s) | -369 | -88 | |
| Zr(s) + ½ $N_2$(g) = ZrN (s) | -365 | -95 | |
| $ZrH_2$(s) + ½ $N_2$(g) = ZrN (s) +$H_2$(g) | -196 | 39 | |
| $HfH_2$(s) + ½ $N_2$(g) = HfN (s) + $H_2$(g) | NA | NA | |
| | | | Carbothermal nitridation |
| $ZrO_2$(s) + 2C (graphite) + ½ $N_2$ = ZrN(s) + 2CO(g) | 511 | 276 | ΔG < 0 at T > 1620 °C |
| $HfO_2$(s) + 2C (graphite) + ½ $N_2$ = HfN(s) + 2CO(g) | 523 | 279 | ΔG < 0 at T > 1680 °C |
| $ZrO_2$(s) + 2C (graphite) + $NH_3$ = ZrN(s) + 2CO(g) + 1.5$H_2$ | 553 | 381 | ΔG < 0 at T > 1180 °C |
| $HfO_2$(s) + 2C (graphite) + $NH_3$ = HfN(s) + 2CO(g) + 1.5$H_2$ | 569 | 378 | ΔG < 0 at T > 1220 °C |
| ZrC(s) + 0.5$N_2$(g) = ZrN(s) + C (graphite) | -168 | -85 | ΔG < 0 at T < 1700 °C |
| HfC(s) + 0.5$N_2$(g) = HfN(s) + C (graphite) | -134 | -80 | ΔG < 0 at T < 1500 °C |

†Enthalpies, entropies and free energies (ΔG) of the reactions are from FactSage 5.4 database (www.factsage.com). ‡For reactions with temperatures of the ΔG sign change not indicated, ΔG is negative for the range of stability of reactants

**Table S2.** Cell parameters for NaCl –type compounds reported in Hf-Zr-C-O-N system.

| Formula | a, Å | ICSD | Year | Authors | Title | Reference |
|---|---|---|---|---|---|---|
| HfC | 4.63078 | 159873 | 2008 | Nakamura, K.; Yashima, M. | Crystal structure of (NaCl)-type transition metal monocarbides M C (M =V, Ti, Nb, Ta, Hf, Zr), a neutron powder diffraction study | Materials Science & Engineering, B: Advanced Functional Solid-State Materials (2008) 148, p69-p72 |
| HfC | 4.637 | 658398 | 1995 | Lengauer, W.; Binder, S.; Aigner, K.; Ettmayer, P.; Guillou, A.; Debuigne, J.; Groboth, G. | Solid state properties of group IVb carbonitrides | Journal of Alloys and Compounds (1995) 217, (1) p137-p147 |
| HfC | 4.65 | 658336 | 1994 | Aigner, K.; Lengauer, W.; Rafaja, D.; Ettmayer, P. | Lattice parameters and thermal expansion of $Ti(C_xN_{1-x})$, $Zr(C_xN_{1-x})$, $Hf(C_xN_{1-x})$ and $TiN_{1-x}$ from 298 to 1473 K as investigated by high-temperature X-ray diffraction | Journal of Alloys and Compounds (1994) 215, (1) p121-p126 |
| HfC | 4.633 | 618016 | 1984 | Valvoda, V. | X-ray Debye temperatures of HfC and alpha-$W_2C$ | Physica Status Solidi A: Applied Research (1984) 83, (2) p123-125 |
| HfC | 4.64 | 618010 | 1977 | Rogl, Peter; Naik, Subhash K.; Rudy, Erwin | A constitutional diagram of the system TiC - HfC - WC | Monatshefte fuer Chemie (1977) 108, (5) p1189-p1211 |
| HfC | 4.635 | 618013 | 1977 | Dubrovskaya, L.B.; Zaytsev, G.P.; Ordanyan, S.S. | Magnetic susceptibility of solid solutions of hafnium and tantalum monocarbides | Physics of Metals and Metallography (1977) 44, (6) p173-p177 |
| HfC | 4.623 | 618008 | 1975 | Constant, K.; Kieffer, R.; Ettmayer, P. | Ueber das pseudoternaere System 'Hf O' - HfN - HfC | Monatshefte fuer Chemie (1975) 106, (4) p973-p981 |
| HfC | 4.623 | 618009 | 1975 | Gatterer, J.; Dufek, G.; Ettmayer, P.; Kieffer, R. | Das kubische Tantalmononitrid (B1-Typ) und seine Mischbarkeit mit den isotypen Uebergangsmetallnitriden und-carbiden | Monatshefte fuer Chemie (1975) 106, (5) p1137-p1147 |
| HfC | 4.674 | 618017 | 1975 | Achour, M.; Zaug, J.; Pialoux, A. | Etude de la carboreduction progressive du dioxyde de hafnium. III. - Determination du parametre cristallin de la phase HfC limite puis sous vide par diffraction des rayons X entre 1000 C et 2000 C | Revue Internationale des Hautes Temperatures et des Refractaires (1975) 12, (3) p289-p294 |
| HfC | 4.623 | 618011 | 1972 | Kieffer, R.; Nowotny, H.; Ettmayer, P.; Dufek, G. | Neue Untersuchungen ueber die Mischbarkeit von Uebergangsmetallnitriden und -karbiden | Metall (Clausthal-Zellerfeld, Germany) (1972) 26, (7) p701-p708 |
| HfC | 4.64 | 617998 | 1968 | Ramqvist, Lars | Variation of lattice parameter and hardness with carbon content of group 4b metal carbides | Jernkontorets Annaler (1968) 152, p 517-523 |
| HfC | 4.631 | 618002 | 1968 | Pessall, N.; Gold, R.E.; Johansen, H.A. | A study of superconductivity in interstitial compounds | Journal of Physics and Chemistry of Solids (1968) 29, (1) p19-p38 |

| Formula | a, Å | ICSD | Year | Authors | Title | Reference |
|---|---|---|---|---|---|---|
| HfC | 4.65 | 618018 | 1967 | Denbnovetskaya, E.N. | Preparation of solid solutions of some complex carbides of the transition metals | Soviet Powder Metallurgy and Metal Ceramics (1967) 6, (3) p194-p197 |
| HfC | 4.643 | 618020 | 1965 | Sara, R.V. | The hafnium-carbon system | Transactions of the Metallurgical Society of AIME (1965) 233, p1683-p1691 |
| HfC | 4.64 | 198351 | 1963 | Krikorian, N.H.; Witteman, W.G.; Bowman, M.G. | The mutual solid solubility of hafnium carbide and uranium monocarbide | Journal of the Electrochemical Society (1963) 110, (6) p560-p563 |
| HfC | 4.6395 | 618000 | 1963 | Krikorian, N.H.; Wallace, T.C.; Anderson, James L. | Low-temperature thermal expansion of the group 4a carbides | Journal of the Electrochemical Society (1963) 110, (6) p587-p588 |
| HfC | 4.632 | 618005 | 1963 | Rudy, E.; Benesovsky, F. | Zur Kenntnis der Stabilitaet des Urandicarbids und der Kohlenstoff-stabilen Bereiche in den Partialsystemen von UC mit ZrC, HfC, NbC und TaC | Monatshefte fuer Chemie (1963) 94, (1) p204-p224 |
| HfC | 4.628 | 618015 | 1961 | Nowotny, H.; Laube, E. | Die thermische Ausdehnung hochschmelzender Phasen | Planseeberichte fuer Pulvermetallurgie (1961) 9, p54-p59 |
| HfC | 4.628 | 108134 | 1960 | Benesovsky, F.; Rudy, E. | Beitrag zum Aufbau des Systeme Zirkonium-Kohlenstoff und Hafnium-Kohlenstoff | Planseeberichte fuer Pulvermetallurgie (1960) 8, p66-p71 |
| HfC | 4.63 | 618004 | 1960 | Nowotny, H.; Benesovsky, F.; Rudy, E. | Hochschmelzende Systeme mit Hafniumkarbid und -nitrid | Monatshefte fuer Chemie (1960) 91, (2) p348-p356 |
| HfC | 4.64 | 618003 | 1959 | Nowotny, H.; Kieffer, R.; Benesovsky, F.; Brukl, C. | Ueber die Teilsysteme: TiC - HfC und ZrC - HfC | Monatshefte fuer Chemie (1959) 90, (1) p86-p88 |
| HfC | 4.641 | 22262 | 1954 | Cotter, P.G.; Kohn, J.A. | Industrial diamond substitutes: I. Physical and x-ray study of hafnium carbide. | Journal of the American Ceramic Society (1954) 37, p415-p420 |
| HfC | 4.64 | 618001 | 1953 | Glaser, Frank W.; Moskowitz, David; Post, Benjamin | A study of some binary hafnium compounds | Journal of Metals (1953) 5, p1119-p1120 |
| **$HfC_{1-x}$** | | | | | | |
| $HfC_{0.901}$ | 4.638 | 43371 | 1962 | Bittner, H.; Goretzki, H. | Magnetische Untersuchungen der Carbide TiC, ZrC, HfC, VC, Nb C und TaC | Monatshefte fuer Chemie (1962) 93, p1000-p1004 |
| $HfC_{0.95}$ | 4.648 | 618007 | 1971 | Samsonow, G.W.; Morosow, W.W. | Carbohydride der Uebergangsmetalle | Monatshefte fuer Chemie (1971) 102, (6) p1667-p1678 |
| $HfC_{0.96}$ | 4.6393 | 617997 | 1970 | Samsonov, G.V.; Timofeeva, I.I. | X-ray diffraction study of dynamic characteristics of crystal lattices of some interstitial phases | Dopovidi Akademii Nauk Ukrains'koi RSR, Seriya A: Fiziko-Tekhnichni ta Matematichni Nauki (1970) 1970, (9) p831-p833 |

| Formula | a, Å | ICSD | Year | Authors | Title | Reference |
|---|---|---|---|---|---|---|
| $HfC_{0.71}$ | 4.627 | | 1991 | Turchanin, A. G.; Turchanin, M. A., | Thermodynamics of Refractory Carbides and Carbonitrides. | Metallurgiya: 1991; p 352 (in Russian) |
| $HfC_{0.85}$ | 4.637 | | | | | |
| $HfC_{0.99}$ | 4.642 | | | | | |
| $HfC_{0.97}$ | 4.6424 | | 1974 | G. V. Samsonov, G. S. Upadhyaya, and V. S. Neshpor | Physical Materials Science of Carbides (in Russian) | Naukova Dumka Kiev, (1974). |
| $HfC_{0.96}$ | 4.6422 | | | | | |
| $HfC_{0.95}$ | 4.6420 | | | | | |
| $HfC_{0.94}$ | 4.6418 | | | | | |
| $HfC_{0.93}$ | 4.6416 | | | | | |
| $HfC_{0.92}$ | 4.6414 | | | | | |
| $HfC_{0.91}$ | 4.6411 | | 1974 | G. V. Samsonov, G. S. Upadhyaya, and V. S. Neshpor | Physical Materials Science of Carbides (in Russian) | Naukova Dumka Kiev, (1974). |
| $HfC_{0.90}$ | 4.6408 | | | | | |
| $HfC_{0.89}$ | 4.6405 | | | | | |
| $HfC_{0.88}$ | 4.6401 | | | | | |
| $HfC_{0.87}$ | 4.6397 | | | | | |
| $HfC_{0.86}$ | 4.6393 | | | | | |
| $HfC_{0.85}$ | 4.6389 | | 1974 | G. V. Samsonov, G. S. Upadhyaya, and V. S. Neshpor | Physical Materials Science of Carbides (in Russian) | Naukova Dumka Kiev, (1974). |
| $HfC_{0.84}$ | 4.6385 | | | | | |
| $HfC_{0.83}$ | 4.6380 | | | | | |
| $HfC_{0.82}$ | 4.6376 | | | | | |
| $HfC_{0.81}$ | 4.6372 | | | | | |
| $HfC_{0.80}$ | 4.6377 | | | | | |
| $HfC_{0.79}$ | 4.6363 | | 1974 | G. V. Samsonov, G. S. Upadhyaya, and V. S. Neshpor | Physical Materials Science of Carbides (in Russian) | Naukova Dumka Kiev, (1974). |
| $HfC_{0.78}$ | 4.6358 | | | | | |
| $HfC_{0.77}$ | 4.6354 | | | | | |
| $HfC_{0.76}$ | 4.6348 | | | | | |
| $HfC_{0.75}$ | 4.6344 | | | | | |
| $HfC_{0.74}$ | 4.6338 | | | | | |
| $HfC_{0.73}$ | 4.6334 | | 1974 | G. V. Samsonov, G. S. Upadhyaya, and V. S. Neshpor | Physical Materials Science of Carbides (in Russian) | Naukova Dumka Kiev, (1974). |
| $HfC_{0.72}$ | 4.6328 | | | | | |
| $HfC_{0.71}$ | 4.6324 | | | | | |
| $HfC_{0.70}$ | 4.6318 | | | | | |
| $HfC_{0.69}$ | 4.6313 | | | | | |
| $HfC_{0.68}$ | 4.6307 | | 1974 | G. V. Samsonov, G. S. Upadhyaya, and V. S. Neshpor | Physical Materials Science of Carbides (in Russian) | Naukova Dumka Kiev, (1974). |
| $HfC_{0.67}$ | 4.6301 | | | | | |
| $HfC_{0.66}$ | 4.6295 | | | | | |

| Formula | a, Å | ICSD | Year | Authors | Title | Reference |
|---|---|---|---|---|---|---|
| $HfC_{0.65}$ | 4.6290 | | | | | |
| $HfC_{0.64}$ | 4.6284 | | | | | |
| $HfC_{0.63}$ | 4.6278 | | | | | |
| $HfC_{0.62}$ | 4.6271 | | | | | |
| $HfC_{0.61}$ | 4.6265 | | 1974 | G. V. Samsonov, G. S. Upadhyaya, and V. S. Neshpor | Physical Materials Science of Carbides (in Russian) | Naukova Dumka Kiev, (1974). |
| $HfC_{0.60}$ | 4.6258 | | | | | |
| $HfC_{0.59}$ | 4.6251 | | | | | |
| $HfC_{0.58}$ | 4.6244 | | | | | |
| $HfC_{0.57}$ | 4.6237 | | | | | |
| $HfC_{0.56}$ | 4.6229 | | | | | |
| $HfC_{0.55}$ | 4.6221 | | | | | |
| $HfC_{0.54}$ | 4.6214 | | 1974 | G. V. Samsonov, G. S. Upadhyaya, and V. S. Neshpor | Physical Materials Science of Carbides (in Russian) | Naukova Dumka Kiev, (1974). |
| $HfC_{0.53}$ | 4.6205 | | | | | |
| $HfC_{0.52}$ | 4.6196 | | | | | |
| $HfC_{0.51}$ | 4.6188 | | | | | |
| $HfC_{0.84}O_{0.16}$ | 4.618 | 196637 | 1975 | Constant, K.; Kieffer, R.; Ettmayer, P. | Ueber das pseudoternaere System 'HfO' - HfN - HfC | Monatshefte fuer Chemie (1975) 106, (4) p973-p981 |
| $HfC_{0.39}O_{0.61}$ | 4.603 | 196638 | | | | |
| $HfC_{0.923}O_{0.058}$ | 4.638 | | 1991 | Turchanin, A. G.; Turchanin, M. A., | Thermodynamics of Refractory Carbides and Carbonitrides. | Metallurgiya: 1991; p 352 (in Russian) |
| **Hf-C-N** | | | | | | |
| $HfC_{0.5}N_{0.5}$ | 4.572 | 77009 | 1960 | Nowotny, H.N.; Benesovsky, F.; Rudy, E. | Hochschmelzende Systeme mit Hafniumkarbid und -nitrid | Monatshefte fuer Chemie (1960) 91, p348-p356 |
| $HfC_{0.25}N_{0.75}$ | 4.538 | | | | | |
| $HfC_{0.5}N_{0.5}$ | 4.569 | 618025 | 1975 | Constant, K.; Kieffer, R.; Ettmayer, P. | Ueber das pseudoternaere System 'HfO' - HfN - HfC | Monatshefte fuer Chemie (1975) 106, (4) p973-p980 |
| $HfC_{0.75}N_{0.25}$ | 4.597 | | | | | |
| $HC_{0.5}N_{0.5}$ | 4.573 | 618026 | 1972 | Kieffer, R.; Nowotny, H.; Ettmayer, P.; Dufek, G. | Neue Untersuchungen ueber die Mischbarkeit von Uebergangsmetallnitriden und -karbiden | Metall (Clausthal-Zellerfeld, Germany) (1972) 26, (7) p701-p708 |
| $HfC_{0.5}N_{0.5}$ | 4.6 | 658337 | 1994 | Aigner, K.; Lengauer, W.; Rafaja, D.; Ettmayer, P. | Lattice parameters and thermal expansion of $Ti(C_xN_{1-x})$, $Zr(C_xN_{1-x})$, $Hf(C_xN_{1-x})$ and $TiN_{1-x}$ from 298 to 1473 K as investigated by high-temperature X-ray diffraction | Journal of Alloys and Compounds (1994) 215, (1) p121-p126 |
| $HfC_{0.5}N_{0.5}$ | 4.583 | 658399 | 1995 | Lengauer, W.; Binder, S.; Aigner, K.; Ettmayer, P.; Guillou, A.; Debuigne, J.; Groboth, G. | Solid state properties of group IVb carbonitrides | Journal of Alloys and Compounds (1995) 217, (1) p137-p147 |

| Formula | a, Å | ICSD | Year | Authors | Title | Reference |
|---|---|---|---|---|---|---|
| $HfC_{0.820}N_{0.163}$ | 4.623 | | 1991 | Turchanin, A. G.; Turchanin, M. A., | Thermodynamics of Refractory Carbides and Carbonitrides. | Metallurgiya: 1991; p 352 (in Russian) |
| $HfC_{0.540}N_{0.434}$ | 4.584 | | | | | |
| $HfC_{0.291}N_{0.664}$ | 4.550 | | | | | |
| **Hf-N-O** | | | | | | |
| $HfN_{0.92}O_{0.08}$ | 4.523 | | 1975 | Constant, K Kieffer, R. Ettmayer, P | Ueber das pseudoternaere System 'HfO' - HfN - HfC | Monatshefte fuer Chemie (1975) 106, (4) p973-p980 |
| $HfN_{0.85}O_{0.15}$ | 4.524 | 196635 | | | | |
| $HfN_{0.8}O_{0.2}$ | 4.524 | | | | | |
| $HfN_{0.75}O_{0.25}$ | 4.525 | | | | | |
| $HfN_{0.70}O_{0.30}$ | 4.523 | | | | | |
| $HfN_{0.60}O_{0.40}$ | 4.526 | | | | | |
| $HfN_{0.40}O_{0.60}$ | 4.534 | 196636 | | | | |
| $HfN_{0.80}O_{0.09}$ | 4.534 | | 1991 | Turchanin, A. G.; Turchanin, M. A., | Thermodynamics of Refractory Carbides and Carbonitrides. | Metallurgiya: 1991; p 352 (in Russian) |
| **HfN** | | | | | | |
| HfN | 4.526 | 658397 | 1995 | Lengauer, W.; Binder, S.; Aigner, K.; Ettmayer, P.; Guillou, A.; Debuigne, J.; Groboth, G. | Solid state properties of group IVb carbonitrides | Journal of Alloys and Compounds (1995) 217, (1) p137-p147 |
| HfN | 4.55 | 658335 | 1994 | Aigner, K.; Lengauer, W.; Rafaja, D.; Ettmayer, P. | Lattice parameters and thermal expansion of $Ti(C_xN_{1-x})$, $Zr(C_xN_{1-x})$, $Hf(C_xN_{1-x})$ and $TiN_{1-x}$ from 298 to 1473 K as investigated by high-temperature X-ray diffraction | Journal of Alloys and Compounds (1994) 215, (1) p121-p126 |
| $HfN_{0.95}$ | 4.526 | | 1991 | Turchanin, A. G.; Turchanin, M. A., | Thermodynamics of Refractory Carbides and Carbonitrides. | Metallurgiya: 1991; p 352 (in Russian) |
| $HfN_{1.09}$ | 4.510 | | | | | |
| HfN | 4.516 | 638655 | 1975 | Constant, K.; Kieffer, R.; Ettmayer, P. | Ueber das pseudoternaere System 'HfO' - HfN - HfC | Monatshefte fuer Chemie (1975) 106, (4) p973-p981 |
| HfN | 4.517 | 638656 | 1975 | Gatterer, J.; Dufek, G.; Ettmayer, P.; Kieffer, R. | Das kubische Tantalmononitrid (B1-Typ) und seine Mischbarkeit mit den isotypen Uebergangsmetallnitriden und-carbiden | Monatshefte fuer Chemie (1975) 106, (5) p1137-p1147 |
| HfN | 4.5101 | 53026 | 1972 | Timofeeva, I.I.; Shvedova, L.K. | Microhardness and thermal expansion of transition metal nitrides at 80-300 K | Izvestiya Akademii Nauk SSSR, Neorganicheskie Materialy (1972) 8, p1169-p1170 |
| HfN | 4.516 | 638660 | 1972 | Kieffer, R.; Nowotny, H.; Ettmayer, P.; Dufek, G. | Neue Untersuchungen ueber die Mischbarkeit von Uebergangsmetallnitriden und –karbiden | Metall (Clausthal-Zellerfeld, Germany) (1972) 26, (7) p701-p708 |

| Formula | a, Å | ICSD | Year | Authors | Title | Reference |
|---|---|---|---|---|---|---|
| HfN | 4.526 | 196611 | 1971 | Kieffer, R.; Ettmayer, P.; Petter, F. | Beitrag zur Kenntnis der Systeme Titan -Chrom -Stickstoff, Zirkonium - Chrom - Stickstoff und Hafnium - Chrom – Stickstoff | Monatshefte fuer Chemie (1971) 102, (5) p1182-p1196 |
| HfN | 4.518 | 638657 | 1970 | Rudy, Erwin | The crystal structures of $Hf_3N_2$ and $Hf_4N_3$ | Metallurgical and Materials Transactions B: Processing Science (1970) 1, (5) p1249-p1252 |
| HfN | 4.512 | 638650 | 1968 | Pessall, N.; Gold, R.E.; Johansen, H.A. | A study of superconductivity in interstitial compounds | Journal of Physics and Chemistry of Solids (1968) 29, (1) p19-p38 |
| HfN | 4.52 | 638654 | 1968 | Holleck, H.; Smailos, E.; Thuemmler, F. | Zur Mischkristallbildung in den quasibinaeren Systemen von UN und den Mononitriden der IV A-Gruppe Ti N, Zr N und Hf N | Monatshefte fuer Chemie (1968) 99, (3) p985-p989 |
| HfN | 4.512 | 638649 | 1967 | Yen, C.M.; Toth, L.E.; Shy, Y.M.; Anderson, D.E.; Rosner, L.G. | Superconducting Hc-Jc and Tc measurements in the Nb-Ni-N, Nb-Hf-N, and Nb-V-N ternary systems | Journal of Applied Physics (Melville, NY, United States) (1967) 38, (5) p2268-p2271 |
| HfN | 4.518 | 638652 | 1961 | Rudy, E.; Benesovsky, F. | Untersuchungen in den Systemen: Hafnium - Bor - Stickstoff und Zirkonium - Bor - Stickstoff | Monatshefte fuer Chemie (1961) 92, (2) p415-p441 |
| HfN | 4.52 | 638651 | 1960 | Nowotny, H.; Benesovsky, F.; Rudy, E. | Hochschmelzende Systeme mit Hafniumkarbid und -nitrid | Monatshefte fuer Chemie (1960) 91, (2) p348-p356 |
| HfN | 4.52 | 76129 | 1953 | Glaser, Frank W.; Moskowitz, David; Post, Benjamin | A study of some binary hafnium compounds | Journal of Metals (1953) 5, p1119-p1120 |
| HfN | 4.682 | 53025 | 1971 | Zainulin, Yu.G.; Alyamovskii, S.I.; Shveikin, G.P.; Gel'd, P.V. | Coefficients of thermal expansion of cubic (NaCl-type) oxycarbides and oxynitrides of zirconium and hafnium | Tepolfizika Vysokikh Temperatur (1971) 9, p496-p499 |
| **ZrC** | | | | | | |
| ZrC | 4.694 | 619157 | 1950 | Duwez, Pol; Odell, Francis | Phase relationships in the binary systems of nitrides and carbides of zirconium, columbium, titanium, and vanadium | Journal of the Electrochemical Society (1950) 97, (10) p299-p304 |
| ZrC | 4.683 | 44730 | 1956 | Samsonov, G.V.; Rozinova, N.S. | Some physicochemical propertierties of alloys of zirconium with carbon | Izvestiya Sektora Platiny i Drugikh Blagorodnykh Metallov, Institut Obshchei i Neorganicheskoi Khimii, Akademiya Nauk SSSR (1956) 27, (*) p126-p132 |
| ZrC | 4.689 | 619179 | 1956 | Tombrel, F. | Contribution a l'etude du systeme ternaire C Ti - C Zr - C W | Plansee Proceedings, Papers Presented at the Plansee Seminar, "De Re Metallica", 1955, 2nd (1956) 1956, (*) p205-p215 |

| Formula | a, Å | ICSD | Year | Authors | Title | Reference |
|---|---|---|---|---|---|---|
| ZrC | 4.6828 | 44495 | 1958 | Elliott, R.O.; Kempter, C.P. | Thermal expansion of some transition metal carbides | Journal of Chemical Physics (1958) 62, (*) p630-p631 |
| ZrC | 4.696 | 619159 | 1959 | Brownlee, L.D. | The pseudo-binary systems of uranium carbide with zirconium carbide, tantalum carbide, and niobium carbide | Journal of the Institute of Metals (1959) 87, (*) p58-p61 |
| ZrC | 4.694 | 619172 | 1960 | Benesovsky, F.; Rudy, E. | Beitrag zum Aufbau des Systeme Zirkonium-Kohlenstoff und Hafnium-Kohlenstoff | Planseeberichte fuer Pulvermetallurgie (1960) 8, (*) p66-p71 |
| ZrC | 4.687 | 619151 | 1963 | Smith, Deane K.; Cline, Carl F. | An X-ray investigation of polymorphism in ZrC | Journal of the American Ceramic Society (1963) 46, (11) p566-p566 |
| ZrC | 4.6986 | 619158 | 1963 | Krikorian, N.H.; Wallace, T.C.; Anderson, James L. | Low-temperature thermal expansion of the group 4a carbides | Journal of the Electrochemical Society (1963) 110, (6) p587-p588 |
| ZrC | 4.696 | 619166 | 1963 | Rudy, E.; Benesovsky, F. | Zur Kenntnis der Stabilitaet des Urandicarbids und der Kohlenstoff-stabilen Bereiche in den Partialsystemen von U C mit Zr C, Hf C, Nb C und Ta C | Monatshefte fuer Chemie (1963) 94, (1) p204-p224 |
| ZrC | 4.694 | 619178 | 1963 | Ivanov, O.S.; Alekseeva, Z.M. | Investigation of the structure of alloys of the systems U C - Zr C, U C - Th C, AND Th C - Zr C | Stroenie Splavov Nekotorykh Sistem s Uranom i Toriem (1963) 1963, (*) p419-p429 |
| ZrC | 4.6983 | 619152 | 1965 | Sara, R.V. | The system zirconium-carbon | Journal of the American Ceramic Society (1965) 48, (5) p243-p247 |
| ZrC | 4.682 | 619153 | 1966 | Avgustinik, A.I.; Ordan''yan, S.S. | Structure of alloys in the system Zr-C-Ta | Journal of Applied Chemistry of the USSR (1966) 39, (2) p289-p293 |
| ZrC | 4.68 | 619173 | 1967 | Denbnovetskaya, E.N. | Preparation of solid solutions of some complex carbides of the transition metals | Soviet Powder Metallurgy and Metal Ceramics (1967) 6, (3) p194-p197 |
| ZrC | 4.697 | 619156 | 1968 | Ramqvist, Lars | Variation of lattice parameter and hardness with carbon content of group 4b metal carbides | Jernkontorets Annaler (1968) 152, (*) p517-p523 |
| ZrC | 4.69 | 619162 | 1968 | Pessall, N.; Gold, R.E.; Johansen, H.A. | A study of superconductivity in interstitial compounds | Journal of Physics and Chemistry of Solids (1968) 29, (1) p19-p38 |
| ZrC | 4.6983 | 619177 | 1968 | Nickel, Hubertus; Inanc, Oezel; Luecke, Kurt | Beitrag zum System Zirkonium-Kohlenstoff | Zeitschrift fuer Metallkunde (1968) 59, (12) p935-p940 |
| ZrC | 4.693 | 619160 | 1970 | Stuart, H.; Ridley, N. | Thermal expansions of some carbides and tessellated stresses in steel | Journal of the Iron and Steel Institute, London (1970) 208, (*) p1087-p1092 |
| ZrC | 4.694 | 619171 | 1972 | Kieffer, R.; Nowotny, H.; Ettmayer, P.; Dufek, G. | Neue Untersuchungen ueber die Mischbarkeit von Uebergangsmetallnitriden und -karbiden | Metall (Clausthal-Zellerfeld, Germany) (1972) 26, (7) p701-p708 |

| Formula | a, Å | ICSD | Year | Authors | Title | Reference |
|---|---|---|---|---|---|---|
| ZrC | 4.697 | 619148 | 1975 | Bukatov, V.G.; Knyazev, V.I.; Korostin, O.S.; Baranov, V.M. | Temperature dependence of the Young's modulus of metalline carbides | Inorganic Materials (1975) 11, (2) p310-p312 |
| ZrC | 4.694 | 619169 | 1975 | Gatterer, J.; Dufek, G.; Ettmayer, P.; Kieffer, R. | Das kubische Tantalmononitrid (B1-Typ) und seine Mischbarkeit mit den isotypen Uebergangsmetallnitriden und-carbiden | Monatshefte fuer Chemie (1975) 106, (5) p1137-p1147 |
| ZrC | 4.699 | 619170 | 1975 | Constant, K.; Kieffer, R.; Ettmayer, P. | Ueber das pseudoternaere System 'ZrO' - ZrN - ZrC | Monatshefte fuer Chemie (1975) 106, (4) p823-p832 |
| ZrC | 4.692 | 619174 | 1977 | Funke, V.F.; Pshenichnyi, I.V.; Zubarev, P.V.; Pliner, L.A.; Lyakhov, D.M.; Golomazov, V.M. | Some physicomechanical properties of Zr C - Nb C - Mo C1-x alloys | Soviet Powder Metallurgy and Metal Ceramics (1977) 16, (12) p968-p973 |
| ZrC | 4.697 | 619155 | 1978 | Benedict, U.; Richter, K.; Walker, C.T. | Solubility study in the systems PuC-ZrC and PuC-TaC | Journal of the Less-Common Metals (1978) 60, (1) p123-p133 |
| ZrC | 4.699 | 619149 | 1979 | Lesnaya, M.I.; Nemchenko, V.F.; Savin, V.I. | Some properties of solid solutions TiC - ZrC | Inorganic Materials (1979) 15, (1) p61-p64 |
| ZrC | 4.71 | 619147 | 1981 | Matsumoto, Osamu; Yaguchi, Yoichi; Kajiwara, Toshio; Konuma, Mitsuharu; Kanzaki, Yasushi | Preparation and properties of a cubic solid solution in the Mo-Zr-C system | High Temperature Science (1981) 14, (*) p161-p169 |
| ZrC | 4.6896 | 600759 | 1984 | Rahimzadeh, Ebrahim; Joshi, Narayan R.; Singh, Sohan | Thermal expansion of ZrC from 120 to 300 K by an X-ray powder method | Journal of the American Ceramic Society (1984) 67, (7) pC-139-pC-140 |
| ZrC | 4.7 | 619150 | 1987 | Kosukhin, V.V.; Funke, V.F.; Minashkin, V.I.; Smirnov, V.S.; Efremov, Yu.P. | Zirconium nitride and carbonitride coatings obtained by the chemical vapor deposition (CVD) method | Inorganic Materials (1987) 23, (1) p52-p56 |
| ZrC | 4.692 | 658395 | 1995 | Lengauer, W.; Binder, S.; Aigner, K.; Ettmayer, P.; Guillou, A.; Debuigne, J.; Groboth, G. | Solid state properties of group IVb carbonitrides | Journal of Alloys and Compounds (1995) 217, (1) p137-p147 |
| ZrC | 4.69106 | 159874 | 2008 | Nakamura, K.; Yashima, M. | Crystal structure of (NaCl)-type transition metal monocarbides MC (M =V, Ti, Nb, Ta, Hf, Zr), a neutron powder diffraction study | Materials Science & Engineering, B: Advanced Functional Solid-State Materials (2008) 148, (*) p69-p72 |

| Formula | a, Å | ICSD | Year | Authors | Title | Reference |
|---|---|---|---|---|---|---|
| **$ZrC_{1-x}$** | | | | | | |
| $ZrC_{0.85}$ | 4.692 | 619168 | 1971 | Samsonow, G.W.; Morosow, W.W. | Carbohydride der Uebergangsmetalle | Monatshefte fuer Chemie (1971) 102, (6) p1667-p1678 |
| $ZrC_{0.9}$ | 4.6929 | 77575 | 1970 | Samsonov, G.V.; Timofeeva, I.I. | X-ray diffraction study of dynamic characteristics of crystal lattices of some interstitial phases | Dopovidi Akademii Nauk Ukrains'koi RSR, Seriya A: Fiziko-Tekhnichni ta Matematichni Nauki (1970) 1970, (9) p831-p833 |
| $ZrC_{0.9}$ | 4.698 | 57069 | 1990 | Christensen, A.N. | A neutron diffraction investigation on single crystals of titanium oxide, zirconium carbide and hafnium nitride | Acta Chemica Scandinavica (1990) 44, (*) p851-p852 |
| $ZrC_{0.92}$ | 4.689 | 619154 | 1968 | Vil''k, Yu.N.; Danisina, I.N.; Omel''chenko, Y.A.; Ryzhkova, T.P. | Variations of certain physicochemical properties in the system zirconium carbide - zirconium nitride | Journal of Applied Chemistry of the USSR (1968) 41, (4) p873-p875 |
| $ZrC_{0.957}$ | 4.691 | 43370 | 1962 | Bittner, H.; Goretzki, H. | Magnetische Untersuchungen der Carbide TiC, ZrC, HfC, VC, NbC und TaC | Monatshefte fuer Chemie (1962) 93, (*) p1000-p1004 |
| $Zr_{0.35}C_{0.65}$<br>$Zr_{0.615}C_{0.385}$ | 4.698<br>4.6941 | 292096<br>292095 | 1965 | Sara, R.V. | The system zirconium-carbon | Journal of the American Ceramic Society (1965) 48, (5) p243-p247 |
| $ZrC_{0.69}$<br>$ZrC_{0.76}$<br>$ZrC_{0.99}$ | 4.689<br>4.697<br>4.698 | | 1991 | Turchanin, A. G.; Turchanin, M. A., | Thermodynamics of Refractory Carbides and Carbonitrides. (Table 20 p 95) | Metallurgiya: 1991; p 352 (in Russian) |
| $ZrC_{0.97}$<br>$ZrC_{0.96}$<br>$ZrC_{0.95}$<br>$ZrC_{0.94}$<br>$ZrC_{0.93}$<br>$ZrC_{0.92}$<br>$ZrC_{0.91}$ | 4.6975<br>4.6981<br>4.6986<br>4.6991<br>4.6996<br>4.7000<br>4.7004 | | 1974 | G. V. Samsonov, G. S. Upadhyaya, and V. S. Neshpor | Physical Materials Science of Carbides (in Russian) | Naukova Dumka Kiev, (1974). |
| $ZrC_{0.90}$<br>$ZrC_{0.89}$<br>$ZrC_{0.88}$<br>$ZrC_{0.87}$<br>$ZrC_{0.86}$<br>$ZrC_{0.85}$<br>$ZrC_{0.84}$ | 4.7007<br>4.7011<br>4.7013<br>4.7016<br>4.7018<br>4.7019<br>4.7020 | | 1974 | G. V. Samsonov, G. S. Upadhyaya, and V. S. Neshpor | Physical Materials Science of Carbides (in Russian) | Naukova Dumka Kiev, (1974). |

| Formula | a, Å | ICSD | Year | Authors | Title | Reference |
| --- | --- | --- | --- | --- | --- | --- |
| $ZrC_{0.83}$ | 4.7021 | | | | | |
| $ZrC_{0.82}$ | 4.7021 | | | | | |
| $ZrC_{0.81}$ | 4.7021 | | | | | |
| $ZrC_{0.80}$ | 4.7020 | | 1974 | G. V. Samsonov, G. S. Upadhyaya, and V. S. Neshpor | Physical Materials Science of Carbides (in Russian) | Naukova Dumka Kiev, (1974). |
| $ZrC_{0.79}$ | 4.7019 | | | | | |
| $ZrC_{0.78}$ | 4.7018 | | | | | |
| $ZrC_{0.77}$ | 4.7017 | | | | | |
| $ZrC_{0.76}$ | 4.7016 | | | | | |
| $ZrC_{0.75}$ | 4.7014 | | | | | |
| $ZrC_{0.74}$ | 4.7012 | | 1974 | G. V. Samsonov, G. S. Upadhyaya, and V. S. Neshpor | Physical Materials Science of Carbides (in Russian) | Naukova Dumka Kiev, (1974). |
| $ZrC_{0.73}$ | 4.7010 | | | | | |
| $ZrC_{0.72}$ | 4.7008 | | | | | |
| $ZrC_{0.71}$ | 4.7006 | | | | | |
| $ZrC_{0.70}$ | 4.7003 | | | | | |
| $ZrC_{0.69}$ | 4.7000 | | | | | |
| $ZrC_{0.68}$ | 4.6996 | | | | | |
| $ZrC_{0.67}$ | 4.6993 | | | | | |
| $ZrC_{0.66}$ | 4.6988 | | 1974 | G. V. Samsonov, G. S. Upadhyaya, and V. S. Neshpor | Physical Materials Science of Carbides (in Russian) | Naukova Dumka Kiev, (1974). |
| $ZrC_{0.65}$ | 4.6983 | | | | | |
| $ZrC_{0.64}$ | 4.6976 | | | | | |
| $ZrC_{0.63}$ | 4.6965 | | | | | |
| $ZrC_{0.62}$ | 4.6950 | | | | | |
| **Zr-C-N** | | | | | | |
| $ZrC_{0.5}N_{0.5}$ | 4.635 | 77213 | 1975 | Constant, K.; Kieffer, R.; Ettmayer, P. | Ueber das pseudoternaere System ZrO-ZrN-ZrC | Monatshefte fuer Chemie (1975) 106, (*) p823-p832 |
| $ZrN_{0.52}C_{0.48}$ | 4.632 | 196652 | | | | |
| $ZrC_{0.5}N_{0.5}$ | 4.635 | 618441 | 1987 | Kosukhin, V.V.; Funke, V.F.; Minashkin, V.I.; Smirnov, V.S.; Efremov, Yu.P. | Zirconium nitride and carbonitride coatings obtained by the chemical vapor deposition (CVD) method | Inorganic Materials (1987) 23, (1) p52-p56 |
| $Zr_2C_{0.92}N_{0.82}$ | 4.642 | 618442 | 1968 | Vil''k, Yu.N.; Danisina, I.N.; Omel''chenko, Y.A.; Ryzhkova, T.P. | Variations of certain physicochemical properties in the system zirconium carbide - zirconium nitride | Journal of Applied Chemistry of the USSR (1968) 41, (4) p873-p875 |
| $ZrC_{0.5}N_{0.5}$ | 4.638 | 618445 | 1972 | Kieffer, R.; Nowotny, H.; Ettmayer, P.; Dufek, G. | Neue Untersuchungen ueber die Mischbarkeit von Uebergangsmetallnitriden und -karbiden | Metall (Clausthal-Zellerfeld, Germany) (1972) 26, (7) p701-p708 |

| Formula | a, Å | ICSD | Year | Authors | Title | Reference |
| --- | --- | --- | --- | --- | --- | --- |
| $ZrC_{0.5}N_{0.5}$ | 4.66 | 658334 | 1994 | Aigner, K.; Lengauer, W.; Rafaja, D.; Ettmayer, P. | Lattice parameters and thermal expansion of $Ti(C_xN_{1-x})$, $Zr(C_xN_{1-x})$, $Hf(C_xN_{1-x})$ and $TiN_{1-x}$ from 298 to 1473 K as investigated by high-temperature X-ray diffraction | Journal of Alloys and Compounds (1994) 215, (1) p121-p126 |
| $ZrC_{0.5}N_{0.5}$ | 4.636 | 658396 | 1995 | Lengauer, W.; Binder, S.; Aigner, K. et al. | Solid state properties of group IVb carbonitrides | Journal of Alloys and Compounds (1995) 217, (1) p137-p147 |
| $ZrC_{0.78}N_{0.18}$ | 4.680 | | 1991 | Turchanin, A. G.; Turchanin, M. A., | Thermodynamics of Refractory Carbides and Carbonitrides. (Table 81 p. 194) | Metallurgiya: 1991; p 352 (in Russian) |
| $ZrC_{0.62}N_{0.36}$ | 4.668 | | | | | |
| $ZrC_{0.37}N_{0.63}$ | 4.625 | | | | | |
| **Zr-C-O** | | | | | | |
| $ZrC_{0.71}O_{0.29}$ | 4.667 | 196655 | 1975 | Constant, K.; Kieffer, R.; Ettmayer, P. | Ueber das pseudoternaere System 'ZrO' - ZrN - ZrC | Monatshefte fuer Chemie (1975) 106, (4) p823-p832 |
| $ZrC_{0.31}O_{0.69}$ | 4.643 | 196656 | | | | |
| $ZrC_{0.96}O_{0.04}$ | 4.698 | 181839 | 2011 | Gendre, M.; Maitre, A.; Trolliard, G. | Synthesis of zirconum oxycarbide ($ZrC_xO_y$) powders: influence of stoichiometry on densification kinetics during spark plasma sintering and on mechanical properties | Journal of the European Ceramic Society (2011) 31, (13) p2377-p2385 |
| $ZrC_{0.94}O_{0.05}$ | 4.695 | 181840 | | | | |
| $ZrC_{0.89}O_{0.07}$ | 4.688 | 181841 | | | | |
| $ZrC_{0.79}O_{0.13}$ | 4.68 | 181842 | | | | |
| **ZrN** | | | | | | |
| ZrN | 4.585 | 41934 | 1975 | Christensen, A.N. | A neutron diffraction investigation of single crystals of titanium carbide, titanium nitride, and zirconium nitride. | Acta Chemica Scandinavica, Series A: Physical and Inorganic Chemistry (1975) 29, (*) p563-p568 |
| ZrN | 4.63 | 26950 | 1925 | Becker, K.; Ebert, F. | Die Kristallstrukturen einiger binaerer Carbide und Nitride. | Zeitschrift fuer Physik (1925) 31, (*) p268-p272 |
| ZrN | 4.5745 | 44506 | 1958 | Baker, T.W. | The coefficient of thermal expansion of zirconium nitride | Acta Crystallographica (1958) 11, (*) p300-p300 |
| ZrN | 4.5773 | 76530 | 1972 | Timofeeva, I.I.; Shvedova, L.K. | Microhardness and thermal expansion of transition metal nitrides at 80-300 K | Izvestiya Akademii Nauk SSSR, Neorganicheskie Materialy (1972) 8, (*) p1027-p1029 |
| ZrN | 4.5783 | 169453 | 2011 | Yang Xiao; Takeichi, N.; Shida, K.; Tanaka, H.; Kuriyama, N.; Sakai, T. | Novel Mg-Zr-A-H (A=Li, Na) hydrides synthesized by a high pressure technique and their hydrogen storage properties | Journal of Alloys and Compounds (2011) 509, (*) p1211-p1216 |
| ZrN | 4.68 | 180847 | 2011 | Larijani, M.M.; Kiani, M.; Tanhayi, M.; Majdabadi, A. | Characterization of ion beam sputtered ZrN coatings prepared at different substrate temperatures | Crystal Research and Technology (2011) 46, (4) p351-p356 |
| ZrN | 4.65 | 180848 | | | | |
| ZrN | 4.66 | 180849 | | | | |
| ZrN | 4.63 | 180850 | | | | |

| Formula | a, Å | ICSD | Year | Authors | Title | Reference |
|---|---|---|---|---|---|---|
| ZrN | 4.62 | 197016 | 1987 | Kosukhin, V.V.; Funke, V.F.; Minashkin, V.I.; Smirnov, V.S.; Efremov, Yu.P. | Zirconium nitride and carbonitride coatings obtained by the chemical vapor deposition (CVD) method | Inorganic Materials (1987) 23, (1) p52-p56 |
| ZrN | 4.537 | 644881 | 1954 | Schoenberg, N. | The structure of the metallic quaternary phase ZrTaNO | Acta Chemica Scandinavica (1954) 8, (*) p627-p629 |
| ZrN | 4.576 | 644884 | 1974 | Gusev, A.I.; Shveikin, G.P. | Solid solutions in the system ZrN-NbC | Inorganic Materials (1974) 10, (7) p1087-p1089 |
| ZrN | 4.576 | 644885 | 1987 | Kosukhin, V.V.; Funke, V.F.; Minashkin, V.I.; Smirnov, V.S.; Efremov, Yu.P. | Zirconium nitride and carbonitride coatings obtained by the chemical vapor deposition (CVD) method | Inorganic Materials (1987) 23, (1) p52-p56 |
| ZrN | 4.576 | 644887 | 1950 | Duwez, Pol; Odell, Francis | Phase relationships in the binary systems of nitrides and carbides of zirconium, columbium, titanium, and vanadium | Journal of the Electrochemical Society (1950) 97, (10) p299-p304 |
| ZrN | 4.573 | 644888 | 1968 | Pessall, N.; Gold, R.E.; Johansen, H.A. | A study of superconductivity in interstitial compounds | Journal of Physics and Chemistry of Solids (1968) 29, (1) p19-p38 |
| ZrN | 4.58 | 644889 | 1960 | Nowotny, H.; Benesovsky, F.; Rudy, E. | Hochschmelzende Systeme mit Hafniumkarbid und -nitrid | Monatshefte fuer Chemie (1960) 91, (2) p348-p356 |
| ZrN | 4.577 | 644891 | 1961 | Rudy, E.; Benesovsky, F. | Untersuchungen in den Systemen: Hafnium - Bor - Stickstoff und Zirkonium - Bor - Stickstoff | Monatshefte fuer Chemie (1961) 92, (2) p415-p441 |
| ZrN | 4.558 | 644892 | 1963 | Bittner, H.; Goretzki, H.; Benesovsky, F.; Nowotny, H. | Ueber einige Monocarbid-Mononitrid-Systeme und deren magnetische Eigenschaften | Monatshefte fuer Chemie (1963) 94, (3) p518-p526 |
| ZrN | 4.577 | 644893 | 1968 | Holleck, H.; Smailos, E.; Thuemmler, F. | Zur Mischkristallbildung in den quasibinaeren Systemen von UN und den Mononitriden der IV A-Gruppe Ti N, Zr N und HfN | Monatshefte fuer Chemie (1968) 99, (3) p985-p989 |
| ZrN | 4.568 | 644894 | 1971 | Kieffer, R.; Ettmayer, P.; Petter, F. | Beitrag zur Kenntnis der Systeme Titan -Chrom -Stickstoff, Zirkonium - Chrom - Stickstoff und Hafnium - Chrom - Stickstoff | Monatshefte fuer Chemie (1971) 102, (5) p1182-p1196 |
| ZrN | 4.571 | 644895 | 1975 | Constant, K.; Kieffer, R.; Ettmayer, P. | Ueber das pseudoternaere System 'ZrO' - ZrN - ZrC | Monatshefte fuer Chemie (1975) 106, (4) p823-p832 |
| ZrN | 4.578 | 644896 | 1975 | Gatterer, J.; Dufek, G.; Ettmayer, P.; Kieffer, R. | Das kubische Tantalmononitrid (B1-Typ) und seine Mischbarkeit mit den isotypen Uebergangsmetallnitriden und-carbiden | Monatshefte fuer Chemie (1975) 106, (5) p1137-p1147 |

| Formula | a, Å | ICSD | Year | Authors | Title | Reference |
| --- | --- | --- | --- | --- | --- | --- |
| ZrN | 4.571 | 644897 | 1972 | Kieffer, R.; Nowotny, H.; Ettmayer, P.; Dufek, G. | Neue Untersuchungen ueber die Mischbarkeit von Uebergangsmetallnitriden und -karbiden | Metall (Clausthal-Zellerfeld, Germany) (1972) 26, (7) p701-p708 |
| ZrN | 4.6 | 658332 | 1994 | Aigner, K.; Lengauer, W.; Rafaja, D.; Ettmayer, P. | Lattice parameters and thermal expansion of $Ti(C_xN_{1-x})$, $Zr(C_xN_{1-x})$, $Hf(C_xN_{1-x})$ and $TiN_{1-x}$ from 298 to 1473 K as investigated by high-temperature X-ray diffraction | Journal of Alloys and Compounds (1994) 215, (1) p121-p126 |
| ZrN | 4.575 | 658394 | 1995 | Lengauer, W.; Binder, S.; Aigner, K.; Ettmayer, P.; Guillou, A.; Debuigne, J.; Groboth, G. | Solid state properties of group IVb carbonitrides | Journal of Alloys and Compounds (1995) 217, (1) p137-p147 |
| **$ZrN_{1-x}$** | | | | | | |
| $ZrN_{0.82}$ | 4.579 | 644886 | 1968 | Vil''k, Yu.N.; Danisina, I.N.; Omel''chenko, Y.A.; Ryzhkova, T.P. | Variations of certain physicochemical properties in the system zirconium carbide - zirconium nitride | Journal of Applied Chemistry of the USSR (1968) 41, (4) p873-p875 |
| $ZrN_{0.92}$ | 4.575 | 644898 | 1985 | Turchanin, A.G.; Babaenko, S.A.; Gusev, A.I.; Marunya, M.S. | Thermodynamic properties of complex carbide nitrides in the quasi-binary ZrN-NbC system at 298-1500 K | Russian Journal of Physical Chemistry (1985) 59, (8) p1136-p1137 |
| $ZrN_{0.99}$ | 4.573 | 644882 | 1970 | Samsonov, G.V.; Timofeeva, I.I. | X-ray diffraction study of dynamic characteristics of crystal lattices of some interstitial phases | Dopovidi Akademii Nauk Ukrains'koi RSR, Seriya A: Fiziko-Tekhnichni ta Matematichni Nauki (1970) 1970, (9) p831-p833 |
| $ZrN_{0.72}$<br>$ZrN_{0.96}$ | 4.577<br>4.570 | | 1991 | Turchanin, A. G.; Turchanin, M. A., | Thermodynamics of Refractory Carbides and Carbonitrides. | Metallurgiya: 1991; p 352 (in Russian) |

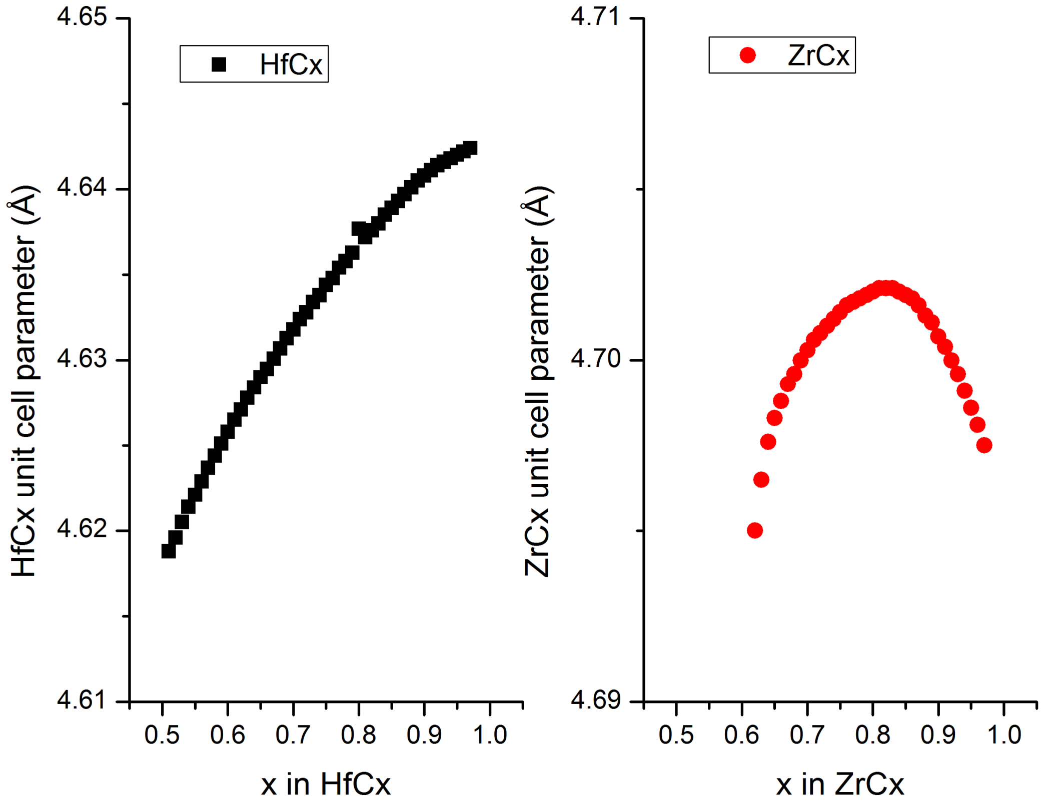


**Figure S1.** Dependence of unit cell parameters of substoichiometric Zr and Hf carbides from carbon content [1].

**Table S3.** Standard enthalpies of formation of $HfC_xO_y$ compounds and their cell parameters measured by Kornilov *et al.* (1977)* using oxygen bomb combustion calorimetry [2].

| Starting materials, synthesis conditions† | (x) C in $HfC_xO_y$ | ± | (y) O in $HfC_xO_y$ | ± | a, Å | $\Delta H°f_{298.15}$ kJ/mol | ± |
|---|---|---|---|---|---|---|---|
| Hf, aceteylene | 0.592 | 0.006 | 0.149 | 0.004 | 4.62 | 200 | 5 |
| T max 1827-1927 °C | 0.697 | 0.006 | 0.117 | 0.003 | 4.62 | 225 | 5 |
| Pressure $10^{-3}$ to $10^{-4}$ Torr | 0.727 | 0.006 | 0.103 | 0.007 | 4.622 | 228 | 5 |
| Hf, carbon black | 0.722 | 0.005 | 0.036 | 0.004 | 4.623 | 195 | 6 |
| T max 2227-2277 °C | 0.763 | 0.006 | 0.033 | 0.003 | (4.624)‡ | 213 | 5 |
| Pressure $10^{-4}$ to $10^{-5}$ Torr | 0.787 | 0.006 | 0.055 | 0.003 | 4.633 | 224 | 6 |
| | 0.801 | 0.006 | 0.015 | 0.003 | (4.632)‡ | 206 | 5 |
| Hf, graphite | 0.86 | 0.006 | 0.008 | 0.003 | 4.638 | 213 | 5 |
| T max 2227-2277 °C | 0.942 | 0.007 | 0.006 | 0.002 | 4.639 | 214 | 5 |
| Pressure $10^{-4}$ to $10^{-5}$ Torr | 0.982 | 0.006 | 0.017 | 0.002 | 4.641 | 215 | 3 |

†The dwell time was reported as several hours for all synthesis conditions.

‡These samples contained the second phase with cell parameters 4.637 and 4.640 Å respectively.

**Table S4.** Standard enthalpies of formation of $ZrN_xO_y$ compounds and their cell parameters measured by Galbraikh et al. (1970) [3] using oxygen bomb combustion calorimetry.

| Starting materials, synthesis conditions | (x) in $ZrN_xO_y$ | (y) in ZrNxOy | a, Å | Hf 298.15 kJ/mol† |
|---|---|---|---|---|
| Zr in $N_2$ or $NH_3$ | 0.77 | 0 | 4.575 | 331 |
| annealing | 0.86 | 0 | 4.574 | 345 |
| for 100 hours | 0.90 | 0.06 | | 398‡ |
| at 600 °C in $N_2$ | 1 | | 4.573 | 369 |

†uncertainties are not reported and taken as 5%.

‡365 kJ/mol after correction for oxygen assuming athermal mixture with Zr oxide.

**Table S5.** Heat capacities of Zr and Hf carbides reported by Turchanin (1991) [4] from high temperature drop calorimetry experiments.

| T/K | Cp, J/mol/K | | | | | | | | | | | |
|---|---|---|---|---|---|---|---|---|---|---|---|---|
| | $ZrC_{0.99}$ | $ZrC_{0.76}$ | $ZrC_{0.69}$ | HfC | $HfC_{0.95}$ | $HfC_{0.90}$ | $HfC_{0.85}$ | $HfC_{0.8}$ | $HfC_{0.75}$ | $HfC_{0.7}$ | $HfC_{0.65}$ | $HfC_{0.6}$ |
| **1200** | 47.36 | 44.27 | 43.39 | 49.93 | 49.34 | 48.76 | 48.17 | 47.58 | 47.00 | 46.41 | 45.83 | 45.24 |
| **1300** | 48.75 | 45.26 | 44.20 | 50.84 | 50.26 | 49.67 | 49.08 | 48.5 | 47.91 | 47.33 | 46.74 | 46.16 |
| **1400** | 50.15 | 46.28 | 45.01 | 51.76 | 51.17 | 50.58 | 50.00 | 49.41 | 48.83 | 48.24 | 47.66 | 47.07 |
| **1500** | 51.54 | 47.31 | 45.82 | 52.67 | 52.08 | 51.5 | 50.91 | 50.33 | 49.74 | 49.16 | 48.57 | 47.98 |
| **1600** | 52.93 | 48.40 | 46.65 | 53.58 | 53.00 | 52.41 | 51.83 | 51.24 | 50.66 | 50.07 | 49.48 | 48.90 |
| **1700** | 54.32 | 49.56 | 47.53 | 54.50 | 53.91 | 53.33 | 52.74 | 52.16 | 51.57 | 50.98 | 50.4 | 49.81 |
| **1800** | 55.71 | 50.81 | 48.49 | 55.41 | 54.83 | 54.24 | 53.66 | 53.07 | 52.48 | 51.90 | 51.31 | 50.73 |
| **1900** | 57.10 | 52.19 | 49.63 | 56.33 | 55.74 | 55.16 | 54.57 | 53.98 | 53.4 | 52.81 | 52.23 | 51.64 |
| **2000** | 58.50 | 53.73 | 51.06 | 57.24 | 56.66 | 56.07 | 55.48 | 54.9 | 54.31 | 53.73 | 53.14 | 52.55 |
| **2100** | 59.89 | 55.45 | 52.97 | 58.16 | 57.57 | 56.98 | 56.40 | 55.81 | 55.23 | 54.64 | 54.05 | 53.47 |
| **2200** | 61.28 | 57.38 | 55.63 | 59.07 | 58.48 | 57.90 | 57.31 | 56.73 | 56.14 | 55.55 | 54.97 | 54.38 |
| **2300** | 62.67 | 59.54 | 59.37 | 59.98 | 59.4 | 58.81 | 58.23 | 57.64 | 57.05 | 56.47 | 55.88 | 55.30 |
| **2400** | 64.06 | 61.94 | 64.60 | 60.9 | 60.31 | 59.73 | 59.14 | 58.55 | 57.97 | 57.38 | 56.8 | 56.21 |
| **2500** | 65.45 | 64.60 | 71.81 | 61.81 | 61.23 | 60.64 | 60.05 | 59.47 | 58.88 | 58.30 | 57.71 | 57.13 |

**Table S6.** Heat capacities of Zr carbonitrides reported by Turchanin (1991) [4] from high temperature drop calorimetry experiments.

| **T/K** | **Cp, J/mol/K** | | | | | | | | | |
|---|---|---|---|---|---|---|---|---|---|---|
| | **ZrN** | **$ZrN_{0.9}C_{0.1}$** | **$ZrN_{0.8}C_{0.2}$** | **$ZrN_{0.7}C_{0.3}$** | **$ZrN_{0.6}C_{0.4}$** | **$ZrN_{0.5}C_{0.5}$** | **$ZrN_{0.4}C_{0.6}$** | **$ZrN_{0.3}C_{0.7}$** | **$ZrN_{0.2}C_{0.8}$** | **$ZrN_{0.1}C_{0.9}$** |
| **1200** | 53.62 | 53.12 | 52.69 | 52.33 | 52.03 | 51.81 | 51.65 | 51.56 | 51.54 | 51.59 |
| **1300** | 54.12 | 53.62 | 53.19 | 52.83 | 52.54 | 52.31 | 52.15 | 52.06 | 52.04 | 52.09 |
| **1400** | 54.61 | 54.11 | 53.68 | 53.31 | 53.02 | 52.79 | 52.64 | 52.55 | 52.53 | 52.57 |
| **1500** | 55.08 | 54.58 | 54.15 | 53.78 | 53.49 | 53.26 | 53.1 | 53.02 | 52.99 | 53.04 |
| **1600** | 55.53 | 55.03 | 54.60 | 54.24 | 53.94 | 53.72 | 53.56 | 53.47 | 53.45 | 53.50 |
| **1700** | 55.98 | 55.48 | 55.05 | 54.69 | 54.39 | 54.17 | 54.01 | 53.92 | 53.9 | 53.95 |
| **1800** | 56.42 | 55.92 | 55.49 | 55.13 | 54.83 | 54.61 | 54.45 | 54.36 | 54.34 | 54.39 |
| **1900** | 56.86 | 56.36 | 55.93 | 55.56 | 55.27 | 55.04 | 54.88 | 54.8 | 54.78 | 54.82 |
| **2000** | 57.29 | 56.79 | 56.36 | 55.99 | 55.70 | 55.47 | 55.32 | 55.23 | 55.21 | 55.25 |

**Table S7.** Heat capacities of Hf carbonitrides reported by Turchanin (1991) [4] from high temperature drop calorimetry experiments.

| T, K | Cp, J/mol/K of $HfN_{(0.93-x)}C_xO_{0.07}$ | | | | | | | | | | |
|---|---|---|---|---|---|---|---|---|---|---|---|
| | x = 0 | x = 0.1 | x = 0.2 | x = 0.3 | x = 0.4 | x = 0.5 | x = 0.6 | x = 0.7 | x =0.8 | x = 0.9 | x = 0.93 |
| 1200 | 53.97 | 52.29 | 50.85 | 49.67 | 48.73 | 48.05 | 47.61 | 47.42 | 47.49 | 47.8 | 48.36 |
| 1300 | 54.23 | 52.54 | 51.11 | 49.93 | 48.99 | 48.3 | 47.87 | 47.68 | 47.74 | 48.06 | 48.62 |
| 1400 | 54.47 | 52.79 | 51.36 | 50.17 | 49.24 | 48.55 | 48.11 | 47.93 | 47.99 | 48.30 | 48.86 |
| 1500 | 54.71 | 53.03 | 51.59 | 50.41 | 49.47 | 49.79 | 48.35 | 48.16 | 48.23 | 48.54 | 49.10 |
| 1600 | 54.94 | 53.26 | 51.82 | 50.64 | 49.70 | 49.02 | 48.58 | 48.39 | 48.46 | 48.77 | 49.33 |
| 1700 | 55.16 | 53.48 | 52.04 | 50.86 | 49.92 | 49.24 | 48.80 | 48.62 | 48.68 | 48.99 | 49.55 |
| 1800 | 55.38 | 53.70 | 52.26 | 51.08 | 50.14 | 49.46 | 49.02 | 48.83 | 48.90 | 49.21 | 49.77 |
| 1900 | 55.6 | 53.91 | 52.48 | 51.29 | 50.36 | 49.67 | 49.24 | 49.05 | 49.11 | 49.42 | 49.99 |
| 2000 | 55.81 | 54.13 | 52.69 | 51.51 | 50.57 | 49.88 | 49.45 | 49.26 | 49.32 | 49.64 | 50.20 |

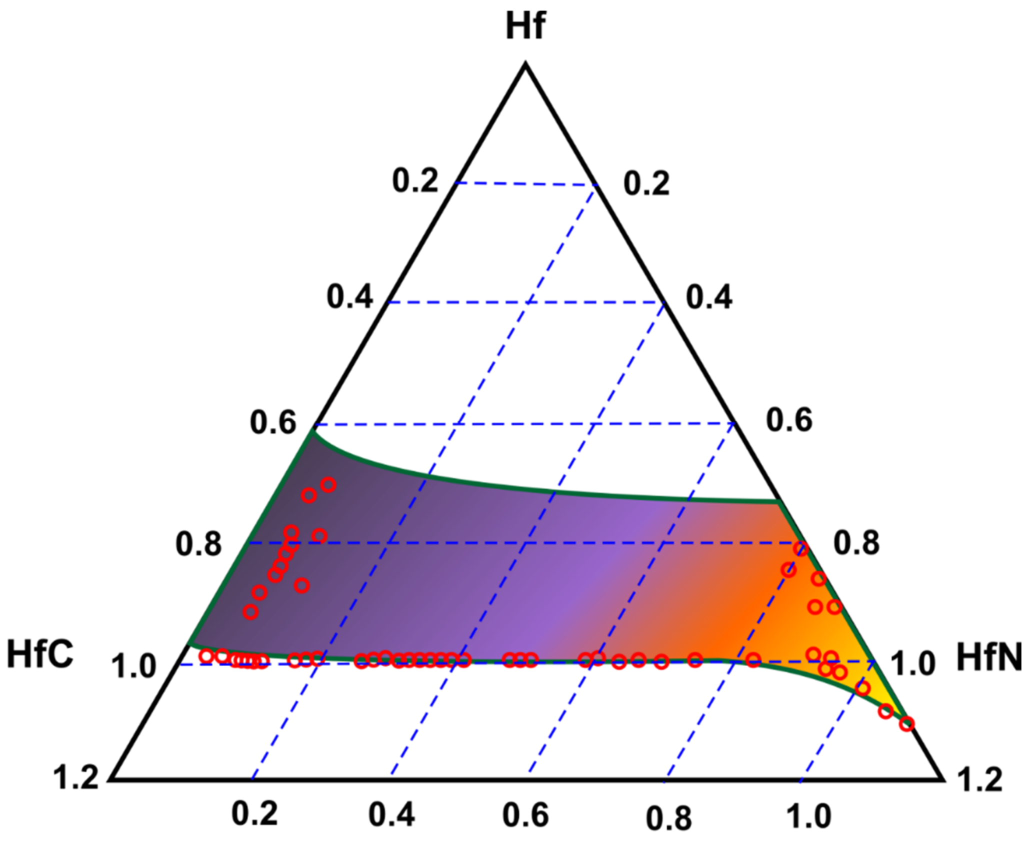


**Figure S2.** Compositions of NaCl-type hafnium carbonitrides in Hf-C-N system at 1500 °C and 1 atm $N_2$. After Brundiers (1975) [5].

## Reference

1. Samsonov, G. V.; Upadhyaya, G. S.; Neshpor, V. S., *Physical Materials Science of Carbides (in Russian)*. Naukova Dumka: Kiev, 1974.
2. Kornilov, A. N.; Chelovskaya, N. V.; Zhelankin, V. I.; Shveikin, G. P., Enthalpies of formation of hafnium carbides. *J. Chem. Thermodyn.* **1977,** 9, (7), 629-42. DOI:10.1016/0021-9614(77)90088-X
3. Gal'braikh, É. I.; Kulik, O. P.; Kuznetsov, A. A.; Lyutaya, M. D.; Morozova, M. P., Enthalpy of formation of the nitrogen solid solution in $\alpha$-zirconium and of zirconium nitride in their homogeneity regions. *Soviet Powder Metallurgy and Metal Ceramics* **1970,** 9, (9), 748-751. DOI: 10.1007/BF00836968
4. Turchanin, A. G.; Turchanin, M. A., *Thermodynamics of Refractory Carbides and Carbonitrides*. Metallurgiya: 1991; p 352 (in Russian)
5. Brundiers, G. D. *Herstellung, Aufbau und Eigenschaften von Hafniumverbindungen im System Hf-C-N-O*; KFK-2161; Germany, 1975; p 278. (Preparation, structure, and properties of hafnium compounds in the system hafnium-carbon-nitrogen-oxygen) http://inis.iaea.org/search/search.aspx?orig_q=RN:07230513
